# Excited State Frequencies of Chlorophyll f and Chlorophyll a and Evaluation of Displacement through Franck-Condon Progression Calculations

**DOI:** 10.3390/molecules24071326

**Published:** 2019-04-04

**Authors:** Noura Zamzam, Jasper J. van Thor

**Affiliations:** Department of Life Sciences, Molecular Biophysics, Imperial College London, London SW7 2AZ, UK; n.zamzam16@imperial.ac.uk

**Keywords:** vibrational frequencies, chlorophyll a, chlorophyll f, excited state, density functional theory, B3LYP, CAM-B3LYP, Franck–Condon

## Abstract

We present ground and excited state frequency calculations of the recently discovered extremely red-shifted chlorophyll f. We discuss the experimentally available vibrational mode assignments of chlorophyll f and chlorophyll a which are characterised by particularly large downshifts of 13^1^-keto mode in the excited state. The accuracy of excited state frequencies and their displacements are evaluated by the construction of Franck–Condon (FC) and Herzberg–Teller (HT) progressions at the CAM-B3LYP/6-31G(d) level. Results show that while CAM-B3LYP results are improved relative to B3LYP calculations, the displacements and downshifts of high-frequency modes are underestimated still, and that the progressions calculated for low temperature are dominated by low-frequency modes rather than fingerprint modes that are Resonant Raman active.

## 1. Introduction

Chlorophylls found in photosynthetic organisms are responsible for light harvesting in the antenna complexes, and the subsequent transfer of excitation energy to photosynthetic reaction centres with almost 100% quantum efficiency. In the reaction centres, specific chlorophylls act as electron transfer cofactors and are involved in the initial charge separation processes [1]. In addition to the recently discovered most red-shifted chlorophyll, Chl f [2], four other chlorophylls are known in oxygenic organisms: chlorophylls a, b, c, and d, with Chl a, having intense absorption maxima at ~ 430 nm and ~ 680 nm, being the most abundant of all [3]. Chlorophyll f, the longest wavelength chlorophyll to date, absorbing between 707 nm in methanol and 800 nm in PSI, can present up to 10% of the chlorophyll composition in certain species of cyanobacteria when grown under far-red light [2,4,5]. The marked difference in the structure of chlorophyll f as compared to that of chlorophyll a is a formyl group replacing the methyl group at the 2^1^ position. This extra carbonyl group in Chl f leads to different vibrational bands in the carbonyl absorption region of the infrared spectrum. In this work, we examine the vibrational properties of chlorophyll a and the newly discovered chlorophyll f in the ground and excited states considering experimental and theoretical data.

Assignment of chlorophyll vibrational bands in experimental spectra: Infrared (IR) absorption spectra of isolated chlorophyll a (in THF solution) in neutral and radical cation states were previously obtained, primarily to assist with the assignment of carbonyl absorption bands observed in the light-induced Fourier transform infrared (FTIR) difference spectra of P700 in photosystem I (PSI) from the cyanobacterium *S. geitleri* [6]. A 1695 cm^−1^ band in the infrared spectrum of neutral chlorophyll a was assigned to the 13^1^-keto (9-keto) carbonyl group while a 1737 cm^−1^ band was assigned to the carbonyl vibration of both 17^3^- and 13^3^-ester (7c- and 10a-ester) groups [6], in line with an earlier assignment of two bands observed at 1695 cm^−1^ and 1735 cm^−1^ in the IR spectra of chlorophyll a in CCl_4_ solution [7]. Prior to that study, similar assignments of a strong 1735 cm^−1^ band to the carbonyl absorption of the two ester groups of chlorophyll a and a strong band around 1700 cm^−1^ to the 13^1^-keto carbonyl were made irrespective of the solvent used [8]. An earlier study by Weigl and Livingston made tentative assignments of a 1740 cm^−1^ band to the 17^3^- and 13^3^-ester groups and a 1700 cm^−1^ band to the 13^1^-keto group based on the comparison of the infrared spectra of chlorophyll a and four related compounds (namely chlorophyll b, pheophytin a, bacteriochlorophyll, and allomerized chlorophyll a) with phytol spectrum [9]. Vibrational bands associated with C=C and C–C modes of chlorophyll porphyrin ring were previously observed in the 1400–1600 cm^−1^ spectral region [6,7,10]. The 13^3^-ester and 13^1^-keto carbonyl bands of chlorophyll a were found to upshift by 13 cm^−1^ and 25 cm^−1^, respectively, upon cation formation [6].

Vibrational band assignments were also established based on early resonance Raman (RR) spectroscopy studies of bacteriochlorophylls and reaction centres. The RR spectrum obtained for chlorophyll a extracted from spinach leaves and dissolved in a liquid crystal system showed four distinct bands in the 1550–1700 cm^−1^ region. Those bands were correlated with similar bands seen at slightly higher frequencies in the RR spectrum of monomer chlorophyll a dissolved in polar solvent [11], and the corresponding assignments of the 1691 cm^−1^ band to stretching vibration of the 13^1^-keto mode and the three other bands at 1555 cm^−1^, 1587 cm^−1^, and 1613 cm^−1^ to ring C=C stretching modes were thus made [12]. It is well established that certain low-frequency modes in photosynthetic chlorins strongly couple to the lowest-energy optical transition and that these frequencies correspond to those observed in time-domain vibrational coherence spectroscopy [13,14,15,16,17,18,19,20,21,22]. However, the vibronic sidebands observed in electronic spectra correspond to finger-print modes including C=C stretching for the Qx transition, and a 1235 cm^−1^ mode dominating the Qy excited Resonance Raman of Ni(II) Pheophytin a, which has been assigned to a C–N stretching mode [23]. Likewise, electronic and fluorescence spectroscopy of chlorophyll a finds typical frequencies of 1525 cm^−1^ and 1145 cm^−1^ from the vibronic bands [24]. High-resolution absorption and fluorescence spectroscopy of porphyrins at low temperature have revealed numerous contributing modes in the 730 cm^−1^ to 1718 cm^−1^ region [25], which were previously evaluated against FC, HT and FCHT calculations [26]. The high-frequency modes are notably underestimated by excited state frequency calculation of displacement. We evaluate such results in this contribution. This is an important consideration for the experimental and computational vibrational mode assignments in the literature, considering the frequency position and intensities observed for excited state finger-print modes [27]. The separation and splitting of the Q band into the Qx and Qy transitions result from the effective asymmetry of the chlorophyll structure such that the effective symmetry is C1 [23]. The Franck–Condon analysis of chlorophyll absorption spectra [28] is commonly treated using the Herzberg–Teller effect [26,29].

Intermolecular interactions strongly influence the carbonyl stretching modes’ frequencies and significant shifts can be induced by factors such as H-bonding or changes in dielectric properties [30]. An RR spectroscopic study probing the conformational changes in the protein environment around bacterial reaction centre pigments associated with charge separation events identified the 13^1^-keto carbonyl modes of reaction centre bacteriochlorophylls at 1683 cm^−1^ (primary donor bacteriochlorophyll pair P), 1685 cm^−1^ and 1689 cm^−1^ (“accessory” bacteriochlorophylls (B) of M and L branches of the RC respectively) in the RR spectra of reaction centres from the purple bacterium *Rhodobacter sphaeroides* [31]. A potential explanation provided for the 14 cm^−1^ downshift observed in the 1689 cm^−1^ band upon P^+^ formation was the formation of H-bond on the “accessory” bacteriochlorophyll of the L (active) branch.

A complete set of assignments of vibrational bands observed in the high-frequency 1425 cm^−1^–1760 cm^−1^ region of the RR spectra of RCs from the purple bacterium while varying the excitation wavelengths for selective excitation of different pigments, aided by calculations of normal coordinates and vibrational frequencies, was provided a few years later [17]. The study assigned 13^1^-keto bands observed at 1989 cm^−1^ and 1693 cm^−1^ to “accessory” bacteriochlorophylls and 1678 cm^−1^ and 1697 cm^−1^ bands to P_M_ and P_L_, respectively. Bands observed at 1746 cm^−1^ and 1738 cm^−1^ were assigned to 13^3^-ester carbonyl modes of “accessory” bacteriochlorophylls and P_L_, respectively, while a double band at 1744 cm^−1^–1750 cm^−1^ was assigned to 13^3^-ester of P_M_ [17]. Near-IR RR spectroscopy studies of RCs from the same purple bacterium [20] and from the green bacterium *Chloroflexus aurantiacus* [21] demonstrated the absence of strong high-frequency modes (including carbonyl modes) in the RR spectra of P or B for Qy resonance excitation. Another study of aggregates of chlorophyll a and BChl c and d pigments also demonstrated the enhanced Raman intensities of low-frequency modes in the Qy-excitation RR spectra [13].

Several infrared spectroscopic experiments performed on the chlorophyll a pairs P700 and P680 in the reaction centres of PSI and photosystem II (PSII), respectively, from different cyanobacteria, also provided chlorophyll mode assignments comparable to those mentioned above. Tavitian et al. (1986) identified a neutral chlorophyll a 13^1^-keto absorption as a 1700 cm^−1^ bleach in light-induced FTIR difference spectra of a green plant PSI film primarily representing P700^+^/P700, but a positive band observed at 1717 cm^−1^ was not yet recognised as the upshifted 13^1^-keto mode associated with chlorophyll a cation formation upon the photooxidation of P700 as deduced later [6,32]. Nevertheless, an upshift in 1734 cm^−1^ and 1748 cm^−1^ ester carbonyl absorption bands to 1742 cm^−1^ and 1753 cm^−1^, respectively, was remarked, which was later rationalised as a consequence of chlorophyll a cation formation [6,32]. Tavitian et al. suggested the assignment of the 1734 cm^−1^(−)/1742 cm^−1^(+) band to the 13^3^-ester group and the 1748 cm^−1^(−)/1753 cm^−1^(+) band to the 17^3^-ester group; however, the assignment of both bands to inequivalent 13^3^-ester carbonyl groups was favoured later, mainly based on comparison with the relative amplitudes of the differential signals of the 13^3^-ester and 13^1^-keto groups in the isolated Chl a^+^ minus Chl a spectrum [6].

Noguchi et al. (1998) reported C=C stretching modes of the neutral chlorophyll macrocycle at 1493 cm^−1^, 1527 cm^−1^, 1555 cm^−1^, and 1610 cm^−1^ in the P680^+^ minus P680 FTIR spectrum measured at 150 K. CC and CN stretching modes and CH bending modes of chlorophyll ring were also observed in the lower frequency region (1100 cm^−1^–1350 cm^−1^). Two bands observed at 1679 cm^−1^ and 1704 cm^−1^ were tentatively assigned to the 13^1^-keto modes of chlorophyll or pheophytin since they also appeared at similar positions in the Resonance Raman spectra [33].

The assignment of 1637 cm^−1^ and 1697 cm^−1^ negative bands in the light-induced P700^+^ minus P700 FTIR spectrum of PSI particles from the cyanobacterium *Synechocystis* obtained at 90 K to 13^1^-keto carbonyl modes of chlorophyll a by Breton et al. was reinforced by the observation of frequency shifts upon isotope labelling [34]. The two bands were downshifted by 2 cm^−1^ upon ^2^H labelling but were not affected by ^15^N labelling which ruled out the possibility of assigning these bands to protein vibrations. The assignments of these bands to 13^1^-keto modes and of two other bands at 1733 cm^−1^ and 1749 cm^−1^ to the 13^3^-ester groups of P700 chlorophylls were further supported by noting that the 13^1^-keto and 13^3^-ester carbonyl bands of isolated chlorophyll a in THF exhibited the same downshifts (3 cm^−1^ and 5 cm^−1^ respectively) upon ^2^H labelling, whereas no ^15^N-induced shifts were observed. The 13^1^-keto and 13^3^-ester bands of P700 chlorophylls were found to upshift upon cation formation [34]. Only two of these bands were downshifted (the 1733 cm^−1^ 13^3^-ester band was downshifted by 5 cm^−1^ and the 1637 cm^−1^ 13^1^-keto was downshifted by 43 cm^−1^) upon the formation of the ^3^P700 triplet state which was considered as evidence of the localisation of triplet state on one of the two chlorophyll a molecules of P700 [34,35].

The chlorophyll carbonyl modes are expected to downshift in the excited state. A downshift by 31 cm^−1^ of the 13^1^-keto mode and by 11 cm^−1^ of the 13^3^-ester carbonyl mode of isolated chlorophyll a in THF upon triplet formation was first reported by Noguchi et al. [36]. The chlorophyll a T_1_ state minus S_0_ state difference FTIR spectrum acted as the basis for the assignment of bands observed in the light-induced FTIR difference spectrum of PSII reaction centre upon triplet formation which showed similar downshifts in bands associated with chlorophyll keto and ester groups [36]. The Chl* minus Chl IR spectrum of chlorophyll a in THF measured by Groot et al. showed a 35 cm^−1^ downshift from 1695 cm^−1^ to 1660 cm^−1^ in the 13^1^-keto band [37]. Downshifted 13^1^-keto bands of chlorophyll in the excited state were observed in the 1620 cm^−1^–1650 cm^−1^ region of the IR spectra of PSII core antenna complexes and reaction centre [37,38,39].

Due to the complexity of biological systems, their infrared difference spectra are usually overcrowded, making their interpretation and the precise assignments of vibrational modes difficult. Such assignments can be aided by theoretical calculations which can be particularly useful in predicting and quantifying frequency shifts occurring as a result of specific changes in the system such as excitation and redox reactions.

Previous theoretical studies of chlorophyll a have demonstrated that B3LYP methods can accurately predict frequency shifts in response to isotope labelling, mutation, or cation/anion formation [40,41,42]. In this study, we present the density function theory (DFT) based calculations of vibrational frequencies of chlorophyll a and chlorophyll f in the ground and excited states using the two functionals B3LYP and CAM-B3LYP, and the 6-31G(d) basis set. CAM-B3LYP is a hybrid exchange-correlation functional proposed by Yanai et al. in 2004 which used a Coulomb-attenuating method to improve long-range properties relative to B3LYP [43]. The new hybrid functional is believed to provide an enhanced description of excited states, particularly charge transfer excitations. Here, we investigate how well the two methods can predict experimentally observed frequency shifts associated with excited state formation. Additionally, we perform FC and HT progression calculations to evaluate the accuracy of mode displacements predicted.

## 2. Results and Discussion

**Figure 1:**
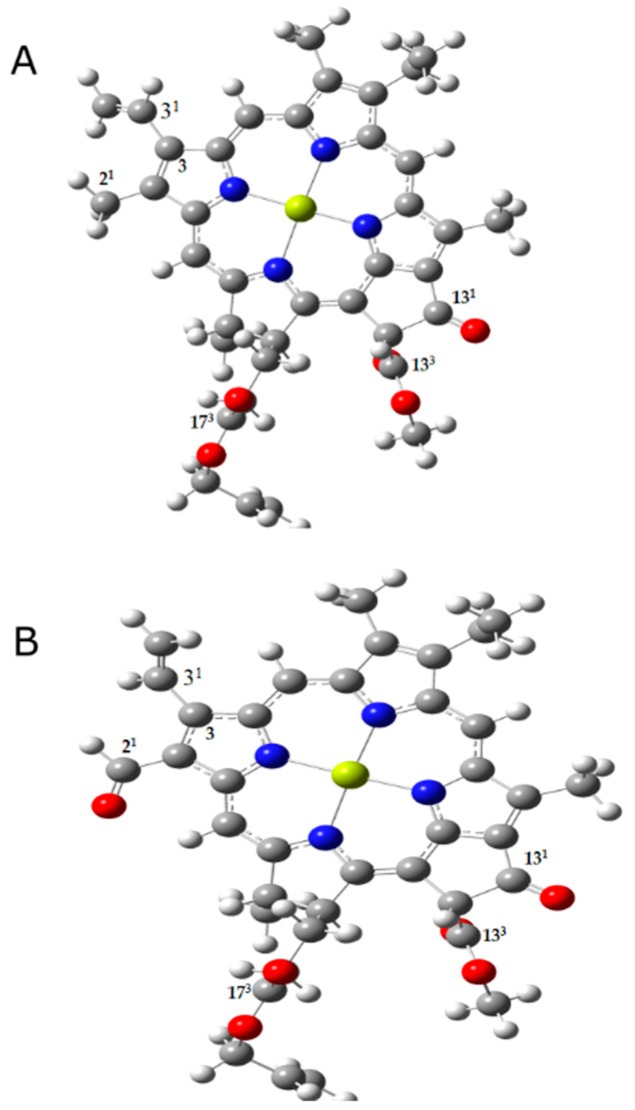
Structures of chlorophyll a (**A**) and chlorophyll f (**B**) models used. Chlorophyll phytyl tail is replaced by CH_2_CH=CH_2_. The carbons associated with carbonyl modes of interest are numbered in the figure according to the IUPAC numbering scheme.

The chlorophyll model used in the calculations closely resembles the structure of chlorophyll, with the single difference being the replacement of the phytyl tail of chlorophyll by CH_2_CH=CH_2_. Our chlorophyll a model (Figure 1A) contains all three carbonyl groups (13^1^-keto, 13^3^ and 17^3^-ester groups) and the chlorophyll f model (Figure 1B) also contains the extra carbonyl group (2^1^-formyl group) [2,44]. The frequency calculations were carried out for the ground and first singlet excited states of chlorophylls a and f, corresponding to the Qy transitions.

The calculated vibrational frequencies are overestimated to a greater extent when using the CAM-B3LYP method. This overestimation is usually corrected by multiplying the frequencies by scaling factors. However, the frequencies presented in Table 1 and Table 2 are unscaled as the application of multiple scaling factors would otherwise be required. No imaginary frequencies were calculated. The total number of vibrational modes calculated for chlorophyll a was 252 and for chlorophyll f was 249 and are all presented in the supplementary material. The modes listed in Table 1 and Table 2 are carbonyl stretching vibrational modes and chlorophyll macrocycle modes in the 1550 cm^−1^–1900 cm^−1^ region. Those modes and associated atomic displacements were individually examined and corresponding assignments were made accordingly. The frequencies calculated using the B3LYP functional are comparable to those previously obtained using the same functional [41,45], with the SVP basis set used in [45]. The two studies provided a complete set of vibrational mode frequencies of chlorophyll a in the ground state, and those of Qy excited state were also provided by Etinski et al. The models of chlorophyll a used in the previous calculations (we refer to Chl-a_4_ of the models presented in [41]) are slightly different from the one we used here. In both models, the chlorophyll phytyl tail was substituted with a methyl group while we had a CH_2_CH=CH_2_ group instead. The methyl and ethyl groups at the 2 and 8 positions in chlorophyll a structure were both replaced by hydrogen molecules in the Chl-a_4_ model in [41]. Therefore, the total number of vibrational modes calculated for chlorophyll a was 186 in [41], 240 in [45], while 252 modes are calculated here due to the larger chlorophyll model we are using, and slight differences in calculated frequencies can be noticed, particularly when considering the carbonyl modes.

The most remarkable difference between chlorophyll a and chlorophyll f modes is the distinct carbonyl vibration mode calculated for chlorophyll f, using both functionals, associated with the additional formyl group characteristic of chlorophyll f (Table 1 and Table 2). This mode appears at the lowest frequency of all C=O vibration modes. Apart from that, all the chlorophyll f modes in both Table 1 and Table 2 are equivalent to the chlorophyll a modes, with slight shifts in frequencies and small differences in intensities. A notable feature in the calculated modes of the two chlorophylls a and f in the ground state using both functionals is the strong coupling between the 13^1^-keto and 13^3^-ester carbonyl stretching modes. This observation, which was also reported in previous DFT calculations of vibrational frequencies of different models of chlorophyll a using B3LYP method and 6-31G(d) basis set [41,46], conflicts with experimental findings where the two modes are uncoupled [6,7,8]. However, in the excited state of both chlorophylls, the coupling between the modes is less pronounced and the 13^3^-ester carbonyl vibration mode is observed at a higher frequency than the 13^1^-keto mode in line with what has been experimentally demonstrated. The theoretical estimation of the frequency downshift expected upon excitation is not accurate. While the frequencies of 13^3^-ester and the 13^1^-keto groups of isolated chlorophyll a have been experimentally shown to downshift by about 11 cm^−1^ and 30 cm^−1^–35 cm^−1^, respectively, upon excitation, the calculated approximation of the downshift in the 13^1^-keto mode does not exceed 26 cm^−1^ with the B3LYP method and reaches 27 cm^−1^ with the CAM-B3LYP method, and is about 5 cm^−1^–8 cm^−1^ in the13^3^-ester C=O mode of the excited states of chlorophylls a and f. As for the 2^1^-formyl group of chlorophyll f, a 17 cm^−1^–23 cm^−1^ downshift in the excited state is predicted. Thus, both B3LYP and CAM-B3LYP methods provided inaccurate estimations of the downshifts in the excited state frequencies of chlorophyll carbonyl modes. The 2^1^-formyl carbonyl mode occurs at a frequency of 1759 cm^−1^ in the B3LYP calculations of the ground state chlorophyll f, which is comparable to the 1763 cm^−1^ frequency previously calculated, at the same level of theory, for the carbonyl mode of the formyl group replacing the vinyl group at the 3^1^ position in chlorophyll d [47]. When a scale factor of 0.94 was applied, the calculated vibrational frequency of 3^1^-formyl carbonyl group of chlorophyll d in the gas phase was found to be close to the 1659 cm^−1^ band assigned to the same group in the Raman spectra of chlorophyll d in acetonitrile reported earlier [47,48].

Vibronic spectra and the Duschinsky effect: The Franck–Condon (FC) approximation ignores the effect of nuclear coordinates on the transition dipole moment for an electronic transition. To account for this, Herzberg–Teller (HT) coupling describes the dependence of the electronic transition on nuclear coordinates. It should include the Duschinsky effect which describes the change in normal coordinates from the initial to the final electronic states and normal mode mixing resulting from the rotation of the potential energy surfaces [29,49,50]. Calculating the spectra of large molecules without taking the Duschinsky rotation into account, thus assuming that the normal modes of the excited state and those of the ground state are identical, could result in major errors in band positions and intensities in high-resolution spectra [49]. The initial electronic (ground) state normal coordinates Q′ are related to the final (excited) state coordinates Q′′ by Q′ = JQ′′ + K according to Duschinsky’s proposal, where J is the Duschinsky matrix describing the normal mode mixing between initial and final states, and K is the displacement vector [49,51]. The electronic transition dipole moment is expressed in terms of nuclear coordinates as a Taylor series expansion:

$$\mu_{e^{\prime },e}=\mu_{0}+\underset{k}{\sum }\mu_{k}Q_{k}+\underset{k,l}{\sum }\mu_{k,l}Q_{k}Q_{l}+\ldots$$

To compute the FC spectrum for strongly allowed transitions, only the zero-order term is taken into account, while the first-order HT terms of the equation must also be considered when computing the FCHT spectrum for weakly-allowed or forbidden transitions [49,51].

**Figure 2:**
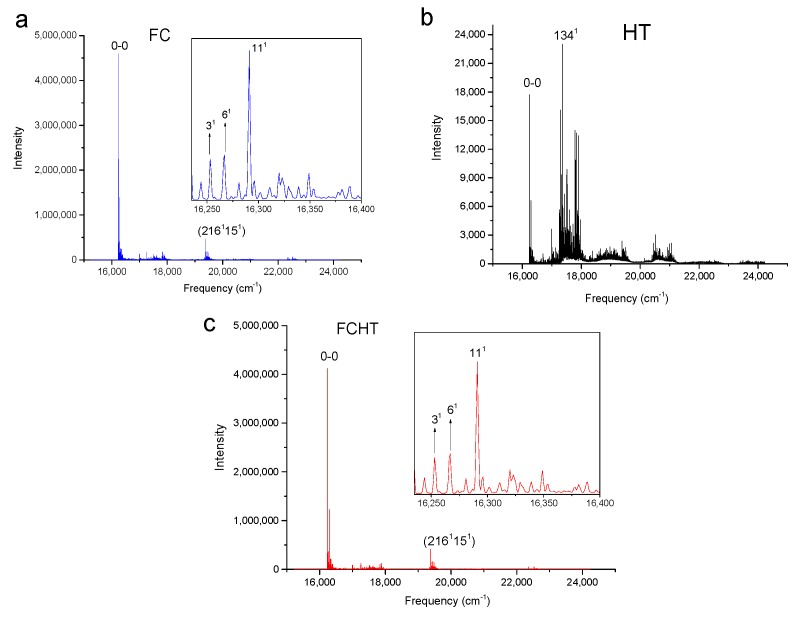
Calculated vibronic absorption spectra of chlorophyll a using the CAM-B3LYP functional and 6-31G(d) basis set, including the Franck–Condon (FC) contribution (**a**), Herzberg–Teller (HT) contribution (**b**), or both FCHT (**c**). The insets magnify specific regions of the spectra associated with assigned vibronic transitions.

**Figure 3:**
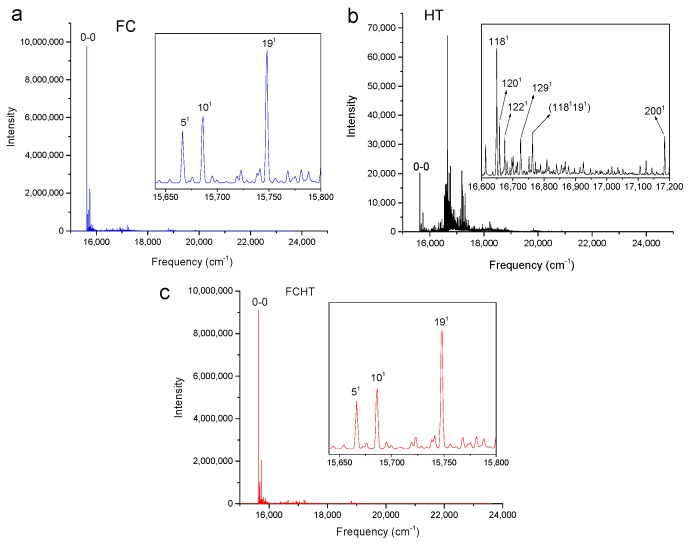
Calculated vibronic absorption spectra of chlorophyll f using the CAM-B3LYP functional and 6-31G(d) basis set, including the FC contribution (**a**), HT contribution (**b**), or both FCHT (**c**). The insets magnify specific regions of the spectra associated with assigned vibronic transitions.

**Figure 4:**
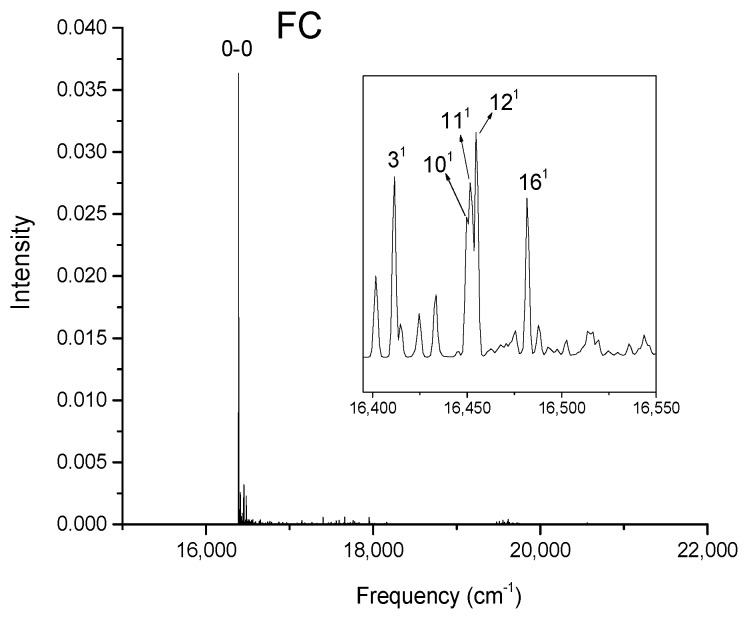
Calculated vibronic absorption spectrum of chlorophyll a using the B3LYP functional and 6-31G(d) basis set, including the FC contribution. The inset magnifies the 16,395–16,550 cm^−1^ region of the spectrum associated with assigned vibronic transitions.

**Figure 5:**
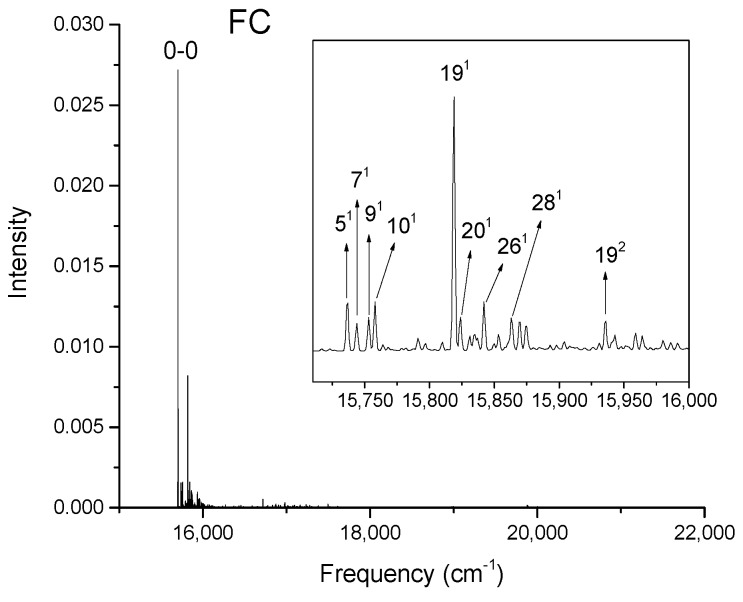
Calculated vibronic absorption spectrum of chlorophyll f using the B3LYP functional and 6-31G(d) basis set, including the FC contribution. The inset magnifies the 15,710–16,000 cm^−1^ region of the spectrum associated with assigned vibronic transitions.

Figure 2, Figure 3, Figure 4 and Figure 5 show the computed vibronic absorption spectra of chlorophylls a and f at T = 0 K. The spectra in Figure 2 and Figure 3 were computed using the CAM-B3LYP functional and 6-31G(d) basis set and include (a) FC contribution, (b) HT contribution, or (c) both FC and HT (FCHT) contributions. The corresponding Duschinsky rotation matrices are presented in the supplementary (Figures S9–S10). Figure 4 and Figure 5 show the spectra computed using the B3LYP functional and 6-31G(d) basis set, including the FC contribution.

The strongest band in the FC spectra of both chlorophylls a and f computed using CAM-B3LYP (Figure 2a and Figure 3a) and B3LYP (Figure 4 and Figure 5) functionals is attributed to the 0-0 transition between ground vibrational levels of the electronic ground and excited states, which clearly dominates the spectra. Only a few side peaks corresponding to transitions to excited vibration levels are computed in the FC spectra. For the FC spectra calculated using the CAM-B3LYP method, those vibronic transitions are assigned to the low-frequency excited normal modes 3^1^, 6^1^, and 11^1^ of chlorophyll a and 5^1^, 10^1^, and 19^1^ of chlorophyll f. An additional combination band appearing in the chlorophyll a spectrum is assigned to 216^1^15^1^ which includes a CH stretching mode (Figure 2a). More vibronic transitions associated with low-frequency excited modes are predicted in the FC spectra of chlorophylls a and f calculated using the B3LYP method with no assignments to mid or high-frequency modes (Figure 4 and Figure 5).

The shape of chlorophyll a and f FCHT spectra computed with the CAM-B3LYP method (Figure 2c and Figure 3c) appear similar to the FC spectra computed with the same method with differences in intensities arising from the HT contribution; the spectra are also dominated by the 0-0 transition and the strongest vibrational bands are the same as those assigned in the FC spectra. However, the shapes of the spectra with only HT contribution (Figure 2b and Figure 3b) for weakly allowed or forbidden transitions not calculated in the FC approximation terms, are markedly different. The 0-0 transition is of much lower intensity and is no longer the strongest band. The strongest bands in the HT spectra of chlorophyll a (Figure 2b) and chlorophyll f (Figure 3b) correspond to the vibronic transitions assigned to the excited state normal modes 134^1^ and 118^1^, respectively, mostly comprising CH bending modes. Additional transition bands in chlorophyll f HT spectrum are assigned to alkene CH bending modes 120^1^, 122^1^, 129^1^, and 118^1^19^1^, with a 200^1^ C=C stretching mode of the chlorophyll macrocycle.

The shift vectors K of normal modes result from the Duschinsky transformation of vibrational normal modes in the electronic excited state from the ground state modes [52]

(1) $$Q^{\prime }=JQ^{\prime \prime }+K$$

where the shift vector *K*

(2) $$K={\left(L^{\prime }\right)}^{-1}M^{1/2}\Delta X$$

which is proportional to the dimensionless displacement *d*

(3) $$d={\left(2\;\pi \;c\frac{\omega^{\prime }}{\hbar }\right)}^{1/2}K$$

and the Duschinsky matrix *J*

(4) $$J={\left(L^{\prime }\right)}^{-1}L^{\prime \prime }$$

where *L’* and *L’’* are the transformation matrices from mass-weighted Cartesian coordinates to normal coordinates. M is the diagonal matrix of atomic masses. *ΔX* is the vector representing the shift in the Cartesian coordinates between initial and final states. The shift vector *K* also provides the intramolecular reorganisation energies, λ

(5) $$\lambda =\frac{1}{2}\underset{i}{\sum }\hbar \omega_{i}K_{i}^{2}$$

**Figure 6:**
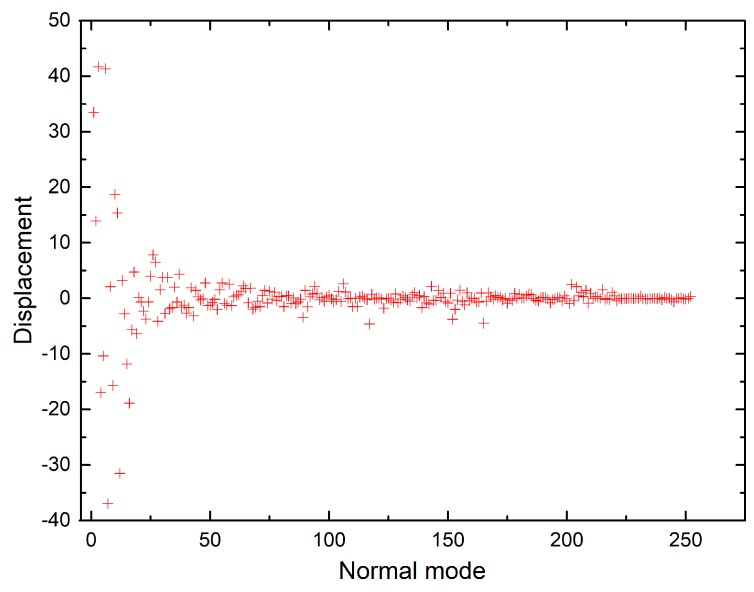
Shift vectors of chlorophyll a normal modes calculated using CAM-B3LYP functional, 6-31G(d) basis set.

**Figure 7:**
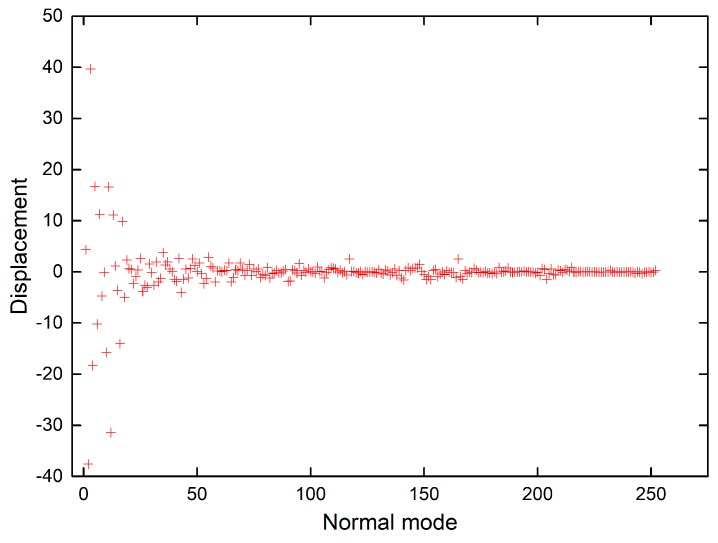
Shift vectors of chlorophyll a normal modes calculated using B3LYP functional, 6-31G(d) basis set.

The shift vectors for electronic excitation of chlorophyll a normal modes calculated using CAM-B3LYP and B3LYP functionals (Tables S9 and S10 in the supplementary) are plotted in Figure 6 and Figure 7. Both plots show that the displacement magnitudes are larger for the low-frequency modes. Additionally, the Duschinsky matrices shown in the supplementary demonstrate that normal mode mixing is distinctly highest for low-frequency modes, hence rationalising the vibronic progressions being generally associated with low-frequency modes as evident in the vibronic absorption spectra (Figure 2, Figure 3, Figure 4 and Figure 5). Although the results show an underestimation in the calculations of the displacements of high-frequency modes, comparing the results of the two different functionals demonstrates improvement in the CAM-B3LYP method and we can clearly notice that it predicts larger displacements of finger-print and skeletal modes than those predicted by B3LYP (Figure 6 and Figure 7).

## 3. Materials and Methods

DFT geometry optimisation and calculation of vibrational frequencies were performed in Gaussian 09, revision A.02 [53] and Gaussian 16, revision A.03 [54] software (Gaussian, Inc., Wallingford, CT, USA). Both B3LYP and CAM-B3LYP functionals were used with the 6-31G(d) basis set for restricted DFT calculations of both the ground and excited states. Following geometry optimisation of ground and excited state structures (equilibrium geometries in Tables S11–S18 in the supplementary material), frequency calculations which did not yield imaginary frequencies were performed and reported in this study (Tables S1–S8 in the supplementary material). The singlet excited state was isolated for optimisation with including 6 states for root 1 and singlets selected only, with both functionals. FC, HT and FCHT calculations were done using the noSymm option and requesting 1 state, ‘JDusch’ calculation and printing all matrices for evaluation, and calculated spectra using ‘SPECHWHM = 1.’, ‘SPECRES = 1′. Gaussview 6 software was used to visualise the calculated vibrational modes and assignments were made correspondingly. This was aided by the assessment of the displacements of the atomic coordinates (provided in the Gaussian output files) associated with vibrational modes of interest (See supplementary Tables S19 and S20 as an illustration).

## 4. Conclusions

We review the assignments of ground and excited states chlorophyll vibrational modes in experimental spectra of isolated chlorophyll a and reaction centres of PSI and PSII, with a particular focus on carbonyl bands shown to downshift upon excited state formation. We also present calculated vibrational frequencies of chlorophyll a and chlorophyll f in the ground and excited states using B3LYP and CAM-B3LYP functionals with the 6-31G(d) basis set. We demonstrate that both functionals predict strong coupling between the 13^1^-keto and 13^3^-ester modes in the ground state which is not seen in the experimental spectra, and that they do not provide accurate estimation of the downshifts in the frequencies of the carbonyl modes in the excited state, particularly the significant downshift of the 13^1^-keto mode illustrated experimentally. By showing the FC, HT, and FCHT absorption spectra at T = 0 K calculated at the CAM-B3LYP/6-31G(d) level, we explain that the calculated vibronic progressions are mostly associated with low-frequency modes showing the strongest displacements. The results additionally show better performance of the CAM-B3LYP method relative to the B3LYP method with regard to the prediction of mode displacements of high-frequency modes known to be vibronic active.

## Figures and Tables

**Table 1:** Vibrational mode frequencies (unscaled) calculated for ground state and excited state chlorophylls a and f using the CAM-B3LYP method and 6-31G(d) basis set, with corresponding mode assignments. Intensities (in km/mol) are shown in parentheses.

Mode	Mode frequencies in cm^−1^ (I)					
	Chl-a			Chl-f		
	Ground State	Excited State	Mode Number	Ground State	Excited State	Mode Number
ν(C=O) 17^3^-ester	1857.97 (317.0833)	1857.41 (315.8875)	214	1856.57 (320.0083)	1856.55 (318.9635)	213
ν_sym_(C=O) 13^1^-keto and 13^3^-ester	1833.18 (211.1643)		213	1837.32 (282.1274)		212
ν(C=O) 13^3^-ester		1828.18 (135.0542) (with a degree of coupling with the 13^1^-keto)			1829.35 (144.0902) (with a degree of coupling with the 13^1^-keto)	
ν_asym_(C=O) 13^1^-keto and 13^3^-ester	1816.05 (792.9019)		212	1821.06 (625.7565)		211
ν(C=O) 13^1^-keto		1806.18 (992.1096) (with a degree of coupling with the 13^3^- ester)			1810.09 (912.2731) (with a degree of coupling with the 13^3^- ester)	
ν(C=O) 2^1^-formyl				1785.68 (521.4323)	1769.05 (541.5630)	210
Vinyl in CH_2_CH=CH_2_ at C17^3^	1753.38 (1.3698)	1753.25 (1.3717)	211	1753.14 (1.3259)	1753.12 (1.2672)	209
Vinyl at C3	1732.52 (17.3765)	1718.97 (96.2622)	210	1732.89 (31.5954)	1719.40 (69.5951)	208
Macrocycle modes	1698.79 (293.0123)	1683.44 (79.6537)	209	1689.63 (397.7097)	1684.46 (26.8216)	207
	1666.44 (51.7727)	1672.73 (264.2016)	208	1665.23 (118.5037)	1668.80 (341.5863)	206
	1663.94 (32.1118)	1649.97 (171.2925)	207	1652.81 (327.7956)	1657.80 (343.2854)	205
	1640.69 (208.8276)	1637.37 (317.9105)	206	1636.42 (253.8413)	1635.44 (494.9683)	204
	1638.92 (500.0061)	1618.55 (148.7429)	205	1616.82 (320.7904)	1609.58 (152.9921)	203
	1610.54 (93.0218)	1604.54 (33.7996)	204	1594.14 (262.3743)	1582.01 (91.3738)	202
	1597.03 (276.4570)	1575.94 (68.8870)	203	1577.34 (21.7559)	1567.39 (84.9900)	201
	1557.01 (106.3392)	1550.06 (91.7621)	202	1556.30 (22.8720)	1551.37 (34.9369)	200

**Table 2:** Vibrational mode frequencies (unscaled) calculated for the ground state and excited state chlorophylls a and f using the B3LYP method and 6-31G(d) basis set, with corresponding mode assignments. Intensities (in km/mol) are shown in parentheses.

Model	Mode frequencies in cm^−1^ (I)					
	Chl-a			Chl-f		
	Ground State	Excited State	Mode Number	Ground State	Excited State	Mode Number
ν(C=O) 17^3^-ester	1844.75 (253.9732)	1844.21 (253.6782)	214	1843.73 (255.4692)	1843.28 (255.377)	213
ν_sym_(C=O) 13^1^-keto and 13^3^-ester	1811.84 (150.7570)		213	1813.68 (166.3241)		212
ν(C=O) 13^3^-ester		1804.93 (75.9686) (with a degree of coupling with the 13^1^-keto)			1806.87 (78.3391) (with a degree of coupling with the 13^1^-keto)	
ν_asym_(C=O) 13^1^-keto and 13^3^-ester	1800.32 (688.1421)		212	1802.54 (619.2428)		211
ν(C=O) 13^1^-keto		1785.77 (959.9775) (with a degree of coupling with the 13^3^-ester)			1790.34 (907.7671) (with a degree of coupling with the 13^3^-ester)	
ν(C=O) 2^1^-formyl				1759.32 (393.4535)	1735.91 (488.9839)	210
Vinyl in CH_2_CH=CH_2_ at C17^3^	1730.33 (1.7880)	1730.20 (1.7956)	211	1730.26 (1.6604)	1730.06 (1.3955)	209
Vinyl at C3	1700.42 (10.0042)	1681.87 (24.5299)	210	1698.60 (25.4969)	1677.08 (81.0752)	208
Macrocycle modes	1654.28 (255.6914)	1639.70 (35.3242)	209	1653.06 (230.3477)	1638.13 (50.2569)	207
	1629.30 (14.1135)	1626.41 (220.3027)	208	1629.98 (17.6638)	1625.80 (168.9423)	206
	1609.28 (54.3863)	1587.20 (138.3399)	207	1613.48 (227.3701)	1599.45 (258.5512)	205
	1602.48 (132.2188)	1577.55 (110.8667)	206	1598.51 (65.9290)	1577.56 (48.6476)	204
	1598.75 (153.5159)	1571.38 (43.2671)	205	1588.30 (162.5164)	1562.40 (120.3871)	203
	1583.42 (100.4916)	1561.76 (5.0663)	204	1563.88 (79.0122)	1545.95 (0.7174)	202
	1568.51 (25.1269)		203	1549.84 (73.0622)		201

## Supplementary Materials

The following are available online, Figures S1–S8: Calculated infrared spectra (mid-frequency and high-frequency regions) for ground and excited states chlorophylls a and f using B3LYP and CAM-B3LYP functionals, Figures S9–S10: Duschinsky matrices correlating the ground and excited states normal modes of chlorophylls a and f calculated using the CAM-B3LYP functional, Tables S1–S8: Calculated frequencies (cm^−1^) of ground and excited states chlorophylls a and f using B3LYP and CAM-B3LYP functionals with 6-31G (d) basis set, Tables S9–S10: Dimensionless displacements (δ) of chlorophyll a normal modes (n) calculated using CAMB3LYP and B3LYP functionals with 6-31G (d) basis set, Tables S11–S18: Cartesian coordinates of ground and excited states chlorophylls a and f using CAM-B3LYP and B3LYP functionals. Tables S19–S20: Displacements of the atomic coordinates associated with carbonyl vibrational modes calculated for chlorophyll a and f in the ground and excited states using CAM-B3LYP method.
