# Supplementary material for: Excited State Frequencies of Chlorophyll f and Chlorophyll a and Evaluation of Displacement through Franck-Condon Progression Calculations
Source: Molecules. 2019 Apr 4;24(7):1326. doi: 10.3390/molecules24071326 (PMC6479460; doi:10.3390/molecules24071326)
Supplement: Supplementary file 1 [file molecules-24-01326-s001.pdf]

molecules

Supplementary Materials

Excited state frequencies of chlorophyll f and chlorophyll a and evaluation of displacement through Franck-Condon progression calculations

Noura Zamzam and Jasper J. van Thor *

Department of Life Sciences, Molecular Biophysics, Imperial College London, London, UK; n.zamzam16@imperial.ac.uk

*Correspondence: j.vanthor@imperial.ac.uk

Received: 16 February 2019; Accepted: 02 April 2019; Published: date

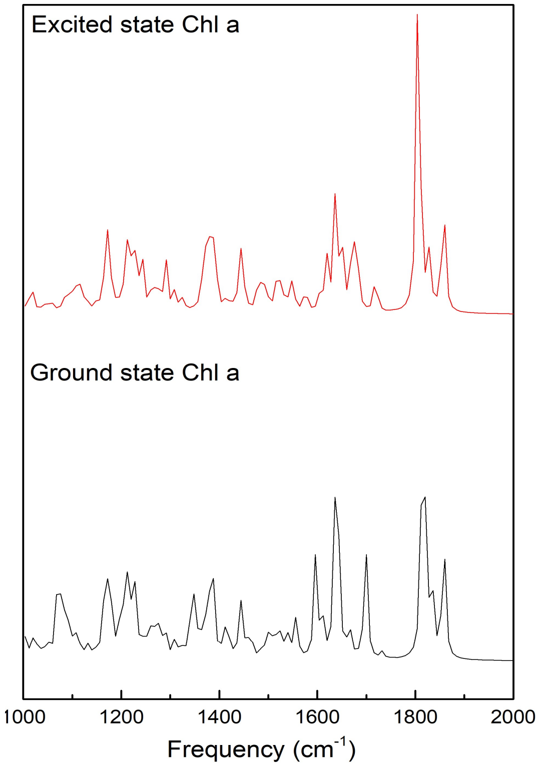

Figure S1. Calculated infrared spectra for ground and excited states of chlorophyll a using CAM-B3LYP functional. The mid-frequency 1000-2000 cm^{-1} region is shown.

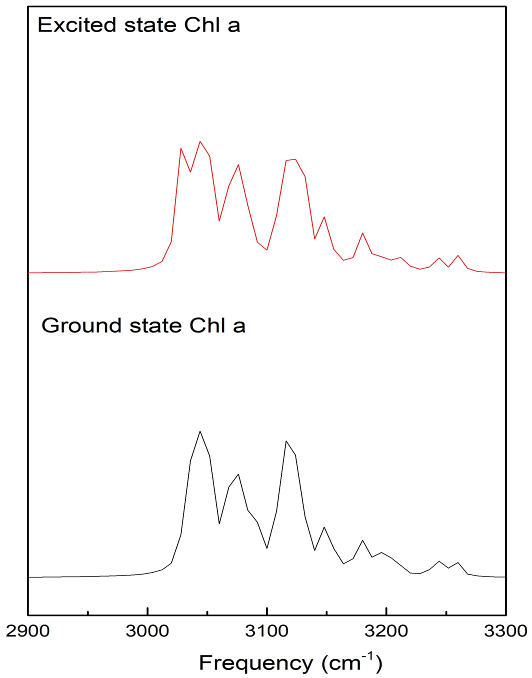

Figure S2. Calculated infrared spectra for ground and excited states of chlorophyll a using CAM-B3LYP functional. The high-frequency 2900-3300 cm^{-1} region is shown.

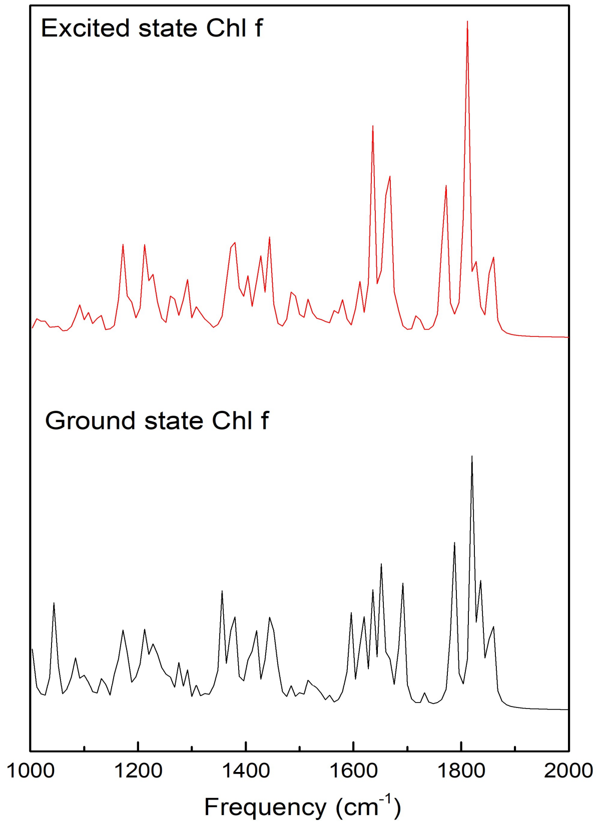

Figure S3. Calculated infrared spectra for ground and excited states of chlorophyll f using CAM-B3LYP functional. The mid-frequency 1000-2000 cm^{-1} region is shown.

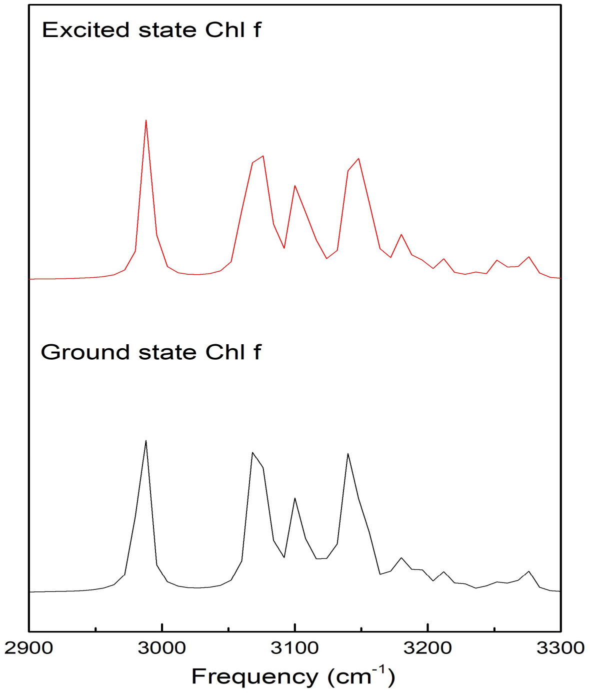

Figure S4. Calculated infrared spectra for ground and excited states of chlorophyll f using CAM-B3LYP functional. The high-frequency 2900-3300 cm^{-1} region is shown.

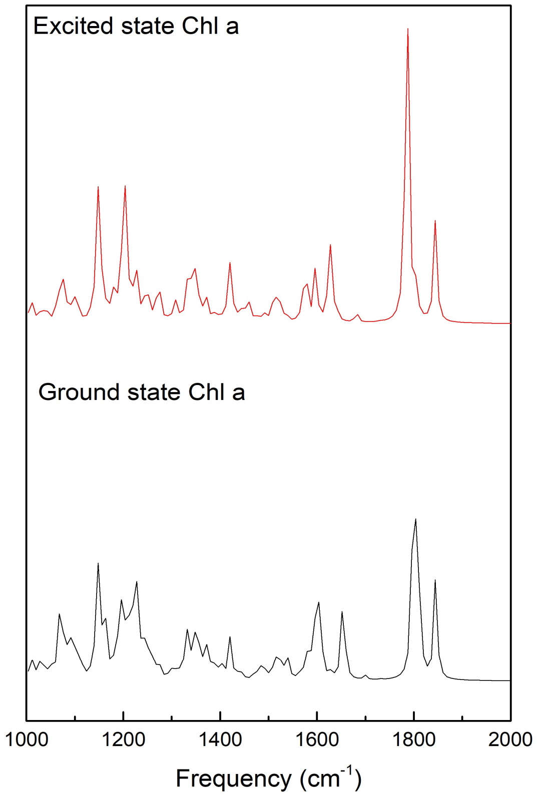

Figure S5. Calculated infrared spectra for ground and excited states of chlorophyll a using B3LYP functional. The mid-frequency 1000-2000 cm^{-1} region is shown.

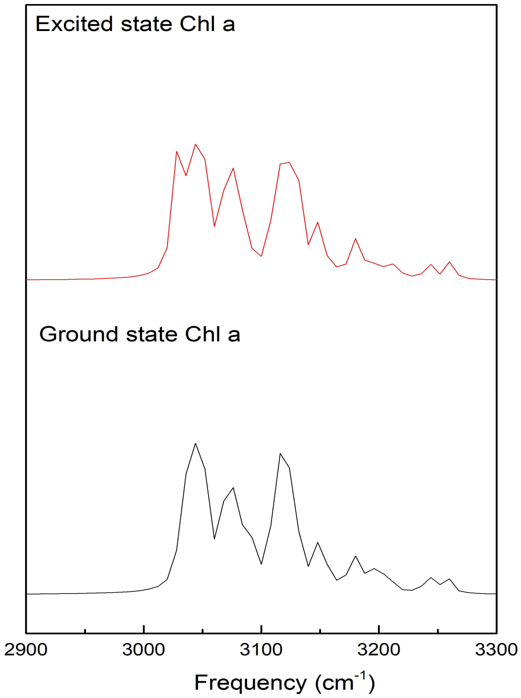

Figure S6. Calculated infrared spectra for ground and excited states of chlorophyll a using B3LYP functional. The high-frequency 2900-3300 cm^{-1} region is shown.

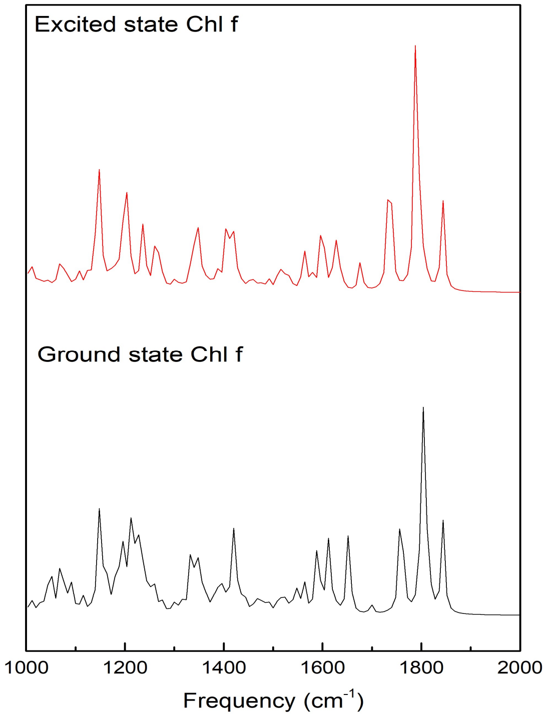

Figure S7. Calculated infrared spectra for ground and excited states of chlorophyll f using B3LYP functional. The mid-frequency 1000-2000 cm^{-1} region is shown.

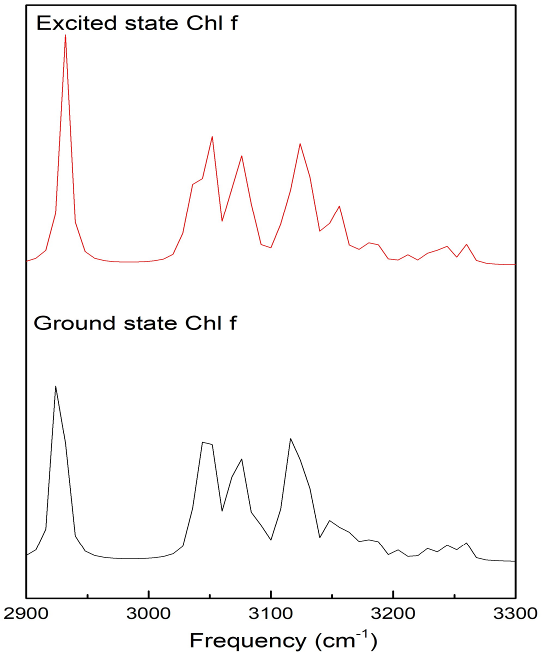

Figure S8. Calculated infrared spectra for ground and excited states of chlorophyll f using B3LYP functional. The high-frequency 2900-3300 cm-1 region is shown.

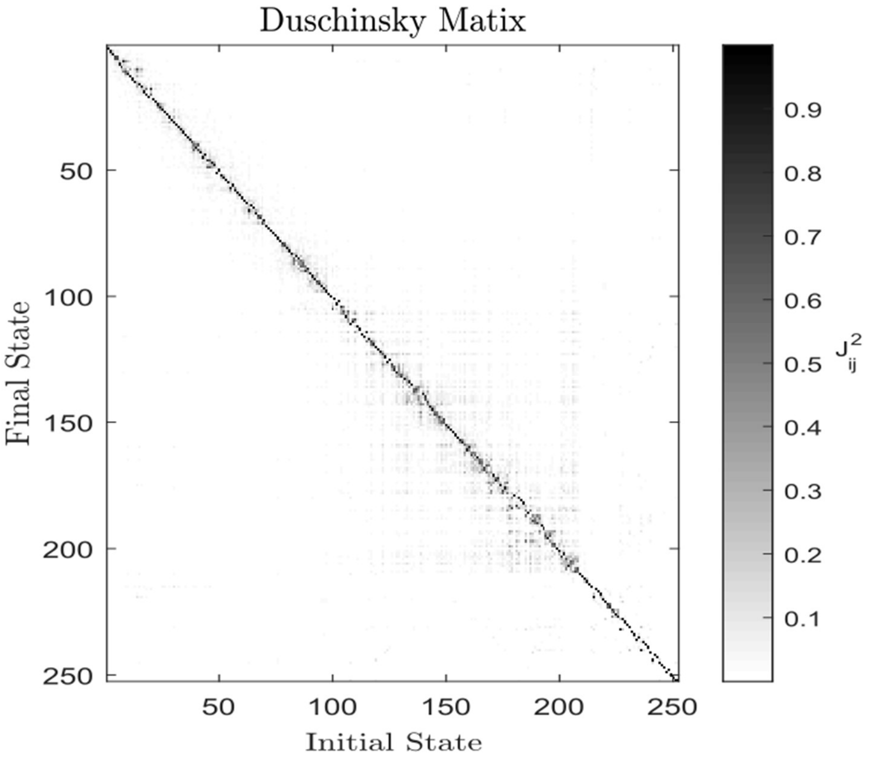

Figure S9. The Duschisky matrix correlating the ground (initial) state and excited (final) state normal modes of chlorophyll a calculated using the CAM-B3LYP functional.

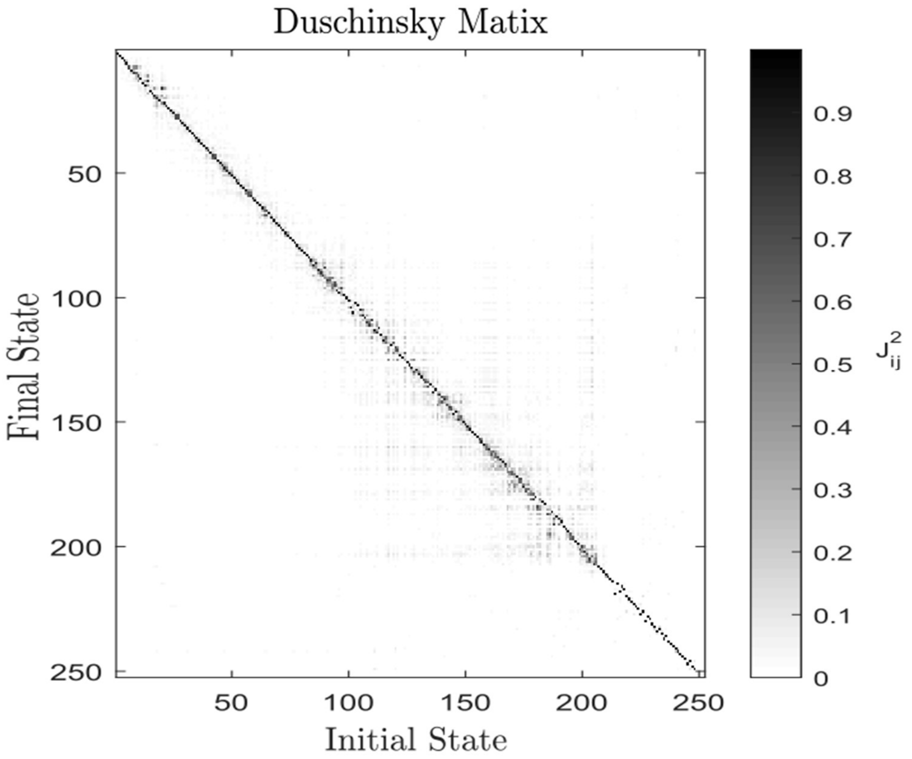

Figure S10. The Duschisky matrix correlating the ground (initial) state and excited (final) state normal modes of chlorophyll f calculated using the CAM-B3LYP functional.

Table S1. Calculated frequencies (cm^{-1}) of ground state chlorophyll a using CAM-B3LYP functional with 6-31G (d) basis set.

10.849	266.222	737.862	1079.824	1413.328	1753.376
13.737	282.1898	741.9342	1092.538	1421.439	1816.052
21.5337	284.8109	750.6164	1095.967	1439.611	1833.184
24.4199	294.4273	758.9692	1103.721	1443.27	1857.97
29.9762	304.1598	763.9984	1109.98	1444.203	3064.32
34.8833	307.4622	765.6368	1113.824	1446.401	3065.468
39.1518	312.4171	780.1567	1130.066	1447.424	3068.022
42.7801	324.8944	784.2532	1144.701	1451.564	3070.63
47.1424	326.6045	792.7317	1151.179	1455.968	3070.732
52.454	351.2187	796.2536	1163.201	1463.881	3071.132
55.5345	355.4323	801.1813	1169.48	1484.479	3076.943
60.5812	363.2308	809.8604	1173.102	1492.664	3078.397
65.474	379.5915	814.0878	1179.671	1497.218	3079.798
70.2177	396.6512	818.8595	1191.433	1498.065	3100.751
79.143	398.3808	853.0659	1192.544	1498.337	3101.056
88.5673	408.939	860.4342	1199.267	1504.958	3102.472
95.7613	416.8129	874.5577	1202.43	1510.596	3105.959
97.0719	421.6852	891.5648	1211.784	1513.312	3106.305
100.612	439.9578	905.8278	1214.685	1518.4	3114.376
115.5303	467.9709	928.8398	1223.313	1518.777	3115.46

118.339	473.1495	930.1869	1225.194	1519.557	3123.413
123.5258	482.5786	936.4732	1229.132	1519.96	3123.555
130.6919	494.1617	939.2747	1243.848	1520.441	3138.832
138.1383	503.3225	943.8531	1247.917	1521.929	3140.538
142.9572	513.1432	960.2733	1258.447	1522.489	3146.162
147.1301	539.1687	961.8967	1272.314	1523.793	3146.274
155.5913	543.2651	970.8006	1288.615	1525.082	3147.306
168.8848	558.9596	980.4914	1309.514	1525.248	3151.952
176.4104	564.6573	982.2719	1311.926	1527.865	3152.277
179.3842	582.6085	995.0534	1324.662	1529.856	3155.006
185.4021	587.4702	999.2081	1333.645	1532.424	3169.381
193.9857	599.5529	1004.786	1336.36	1535.32	3178.166
200.8566	622.4367	1019.665	1344.7	1542.016	3178.99
203.8958	646.5407	1029.965	1351.378	1557.006	3183.005
209.2582	660.6	1037.675	1354.288	1597.033	3197.042
218.3934	674.9298	1042.844	1367.8	1610.536	3201.404
223.5242	688.7546	1048.88	1370.842	1638.919	3211.42
231.1738	694.539	1052.399	1380.982	1640.69	3221.33
243.7971	708.9457	1069.258	1387.188	1663.944	3222.768
245.6519	713.3982	1074.472	1391.723	1666.438	3230.939
248.5335	727.7275	1075.965	1406.462	1698.786	3265.3
255.1628	731.5416	1076.801	1412.795	1732.521	3276.427

Table S2. Calculated frequencies (cm^{-1}) of excited state chlorophyll a using CAM-B3LYP functional with 6-31G (d) basis set.

10.9498	262.805	730.942	1082.432	1413.04	1753.248
13.8646	278.3209	736.7048	1093.605	1419.438	1806.179
20.2346	279.5685	737.9612	1098.683	1426.901	1828.178
24.1432	291.6869	743.0667	1102.931	1441.364	1857.415
32.7376	301.2425	755.2092	1110.716	1442.658	3056.985
33.7717	303.0009	764.8125	1113.213	1442.907	3059.713
40.4819	307.812	774.5284	1121.57	1446.227	3066.605
47.403	323.3732	780.1827	1133.705	1448.561	3068.113
47.9681	323.8072	789.1016	1147.789	1449.854	3069.588
57.5037	347.9688	793.1855	1150.767	1460.107	3072.232
57.9845	352.2847	796.3017	1166.906	1476.085	3074.291

62.6466	360.4847	806.5936	1169.859	1480.406	3077.748
69.4101	377.6971	811.6256	1171.634	1484.15	3081.026
76.6213	386.6321	820.1961	1177.849	1488.19	3099.625
86.8164	391.0995	849.1516	1182.369	1493.185	3100.664
89.6021	402.8078	860.2408	1191.412	1497.279	3101.335
96.1371	417.2271	864.5873	1195.123	1497.921	3102.691
98.5552	420.7061	870.768	1208.1	1511.522	3107.67
111.4654	433.5189	876.466	1213.591	1513.652	3107.718
117.296	461.7842	892.1248	1219.912	1514.467	3114.295
120.3385	470.9264	923.971	1222.84	1515.233	3117.22
123.4601	479.3462	933.0547	1229.67	1515.582	3117.883
130.0527	480.2699	943.1266	1238.757	1517.437	3138.064
138.1627	489.0461	946.5488	1243.409	1519.112	3141.871
143.3749	506.1815	952.6542	1263.91	1519.654	3145.523
148.1583	535.4069	958.3469	1274.078	1521.853	3147.2
155.6138	538.6673	965.4533	1290.406	1524.814	3147.511
167.7606	549.5987	976.0416	1307.652	1525.011	3147.707
173.7786	559.9644	982.0524	1309.663	1525.641	3152.481
179.3063	581.4309	998.3809	1322.253	1527.528	3156.756
186.2263	584.9278	1002.144	1328.055	1528.894	3167.994
192.2762	593.6842	1012.435	1334.915	1533.018	3177.196
197.7318	617.7969	1020.737	1346.741	1540.186	3177.354
202.5731	644.8646	1033.652	1352.272	1550.064	3178.775
207.7165	656.3931	1037.761	1353.935	1575.938	3198.956
215.5933	675.5332	1040.006	1366.265	1604.539	3200.494
222.1813	681.5176	1047.991	1371.336	1618.555	3211.364
229.706	690.6185	1050.28	1377.625	1637.367	3222.508

238.0096	703.1067	1061.291	1381.024	1649.969	3233.719
247.0198	709.079	1070.161	1388.303	1672.734	3238.034
247.8187	720.2227	1072.414	1391.797	1683.438	3264.666
250.4933	729.6551	1072.948	1412.33	1718.966	3278.89

Table S3. Calculated frequencies (cm^{-1}) of ground state chlorophyll f using CAM-B3LYP functional with 6-31G (d) basis set.

11.3551	265.8789	748.9306	1097.9957	1444.355	3069.6225
14.0231	282.4707	755.3953	1103.892	1445.1694	3070.645
21.1784	288.6985	757.6155	1111.4515	1447.6403	3071.4103
24.2792	297.7394	766.1104	1129.1464	1449.7969	3072.1617
31.0756	306.215	772.8332	1136.5603	1452.5359	3076.4477
34.7443	314.4053	784.2401	1150.5707	1461.7254	3078.1184
38.8239	322.5535	786.9274	1159.8603	1481.9113	3086.5219
40.5105	330.9975	795.141	1170.1946	1484.3323	3099.9544
43.1237	347.3739	801.8885	1174.7948	1488.8463	3101.7806
49.6011	354.1088	805.6643	1181.2604	1497.2171	3102.6288
59.6627	358.3571	810.782	1191.4991	1497.9959	3105.727
62.9092	379.4467	815.4549	1192.0581	1503.669	3106.021
67.2028	383.1667	843.1363	1199.7676	1513.0003	3113.7206
70.261	397.7913	863.076	1208.2053	1513.8128	3118.6062
77.1071	403.8061	875.1599	1213.8228	1514.3686	3125.1287
85.0635	406.7953	892.0528	1215.4458	1518.0399	3137.2057
90.0915	418.9776	913.4858	1226.552	1518.6444	3140.4102
95.9012	420.8914	919.9477	1227.9941	1519.1861	3144.9382
110.0846	447.9595	929.3218	1232.969	1519.884	3146.5118
114.3343	457.0593	935.6853	1244.4382	1521.8079	3148.0864
117.5494	480.3961	939.1346	1248.7015	1524.8515	3153.5791
122.4675	488.6951	943.2392	1262.029	1525.6978	3154.5685
127.2332	500.6656	956.0291	1275.9408	1527.583	3172.474
135.4128	508.1606	960.8984	1290.2581	1529.3768	3177.5422
141.3766	523.3371	968.596	1309.9361	1529.8812	3180.4696
142.5886	540.6668	981.924	1314.9428	1532.8696	3186.6974
149.9181	554.3174	984.5611	1325.1351	1542.2649	3196.0115
153.9548	560.7207	986.735	1333.9566	1556.304	3200.0156
168.6775	577.7633	998.4019	1337.5381	1577.3368	3212.8584
175.2397	582.3582	999.077	1347.4683	1594.1363	3225.2885
180.7544	594.1982	1004.234	1354.6515	1616.8216	3241.6456

189.6318	600.8665	1015.7859	1356.6613	1636.4244	3249.8611
193.3928	629.58	1030.3814	1369.0377	1652.8118	3264.3759
202.8683	646.196	1036.0432	1373.3763	1665.2272	3276.2481
205.5816	655.787	1036.9415	1380.7283	1689.6321	
212.9346	682.9765	1045.344	1391.7724	1732.8946	
218.5636	690.6125	1048.2548	1402.1173	1753.1379	
226.3134	699.6658	1052.479	1412.617	1785.6827	
232.0044	708.8569	1068.913	1414.3437	1821.0619	
241.0891	717.2089	1074.6741	1419.748	1837.3186	
247.5464	726.4587	1075.1862	1432.5909	1856.5734	
250.6696	737.3318	1081.274	1439.2607	2985.4174	
263.9159	744.2552	1094.0427	1440.6723	3067.1413	

Table S4. Calculated frequencies (cm^{-1}) of excited state chlorophyll f using CAM-B3LYP functional with 6-31G (d) basis set.

10.6451	256.2605	733.6213	1072.959	1418.01	1810.094
14.0796	263.5383	739.6944	1081.509	1420.016	1829.348
20.7878	279.765	741.3512	1091.638	1426.245	1856.55
24.1769	288.865	751.253	1094.408	1440.691	2989.135
33.1072	295.5596	755.4515	1101.992	1442.118	3061.412
34.3669	305.7311	759.0388	1109.976	1443.174	3066.852
39.1987	306.3387	765.8369	1118.661	1443.687	3071.021
42.5249	320.0223	782.7617	1128.497	1446.873	3071.426
47.6451	324.5182	783.8972	1145.062	1450.051	3073.196
52.5666	344.1878	793.5421	1149.899	1460.108	3076.452
61.6269	352.1567	796.5472	1167.77	1473.402	3080.144
62.3813	356.4044	805.8775	1170.355	1482.074	3083.386
70.1912	376.1601	814.2811	1175.519	1484.349	3100.499
75.4921	381.8445	818.8853	1181.719	1487.048	3101.525
84.5372	387.5846	844.4598	1188.516	1492.454	3102.659
89.5754	394.4286	862.1955	1191.97	1497.465	3105.066
95.2117	405.7625	871.6223	1200.497	1498.06	3109.55
107.8309	417.8308	873.301	1210.08	1510.56	3110.136
114.538	420.4814	886.2821	1214.016	1513.49	3114.282
117.7117	440.1227	899.2032	1221.869	1513.812	3117.542
121.8486	455.399	903.2817	1223.155	1515.267	3139.845
122.8315	477.267	923.1503	1230.475	1515.576	3143.414
128.3699	482.0339	934.6981	1234.616	1517.149	3146.741
134.6757	485.5301	943.4736	1245.264	1519.029	3147.813
139.6474	496.2004	950.028	1263.748	1521.658	3149.439
142.2178	515.1771	962.6937	1281.751	1525.462	3157.299
151.7348	538.831	966.904	1292.384	1525.787	3157.86
154.8265	544.9626	972.7583	1309.416	1528.262	3169.114
167.9592	560.1248	980.2215	1314.809	1528.684	3177.562
173.3504	576.1913	982.3038	1322.97	1532.92	3180.159
178.4508	582.2755	998.3704	1331.902	1540.47	3184.018
187.7389	588.2023	1003.063	1335.305	1551.372	3195.727
191.9617	595.8818	1013.221	1350.207	1567.391	3200.191
200.343	627.4004	1015.547	1352.728	1582.012	3212.825
204.6974	646.5526	1023.418	1354.577	1609.579	3234.017
210.7923	653.8816	1024.868	1366.477	1635.441	3252.283
215.935	680.8616	1035.548	1371.397	1657.803	3256.397

224.4967	685.5404	1040.933	1379.979	1668.796	3264.541
231.7442	697.3553	1048.218	1382.935	1684.46	3276.995
238.5202	704.1353	1051.821	1391.166	1719.4	
243.3594	710.7256	1063.826	1402.211	1753.123	
250.9561	722.2969	1070.558	1412.626	1769.048	

Table S5. Calculated frequencies (cm^{-1}) of ground state chlorophyll a using B3LYP functional with 6-31G (d) basis set.

10.75	265.2909	726.4054	1068.446	1401.271	1730.332
11.2252	278.2743	732.5541	1068.674	1402.643	1800.324
20.7687	279.3731	735.2677	1078.31	1420.069	1811.841
24.5037	293.4905	741.959	1087.767	1423.38	1844.751
34.2307	299.5104	751.1639	1092.344	1435.186	3032.674
41.4108	304.654	757.0911	1103.156	1437.486	3037.217
42.1614	306.8022	764.1563	1112.925	1437.987	3037.596
48.1679	321.1632	773.1982	1134.38	1439.832	3040.646
49.7486	322.9572	780.2767	1142.149	1442.925	3045.947
56.6052	346.2357	782.612	1147.346	1445.556	3047.176
59.4481	350.6508	788.8274	1156.096	1468.758	3048.333
60.714	357.1853	799.5865	1159.718	1474.235	3050.177
66.8592	376.0625	803.6785	1164.426	1478.322	3062.499
71.2066	389.375	809.8816	1178.151	1482.683	3065.522
78.3044	392.3331	843.6095	1181.05	1487.094	3066.855
90.8991	400.3352	845.7435	1185.397	1491.34	3073.686
96.2742	411.5948	856.8979	1190.176	1495.204	3075.061
96.5397	415.1241	874.3435	1196.23	1496.148	3077.199
104.6843	432.7483	879.4456	1197.259	1510.835	3080.253
115.2636	460.5783	886.8293	1207.582	1513.303	3084.656
119.3657	467.578	913.4371	1213.215	1515.389	3087.995
123.5698	475.8333	916.6562	1220.517	1516.007	3091.634
129.3096	487.7942	931.2095	1228.776	1516.4	3108.723
132.2451	493.6997	933.0148	1233.26	1517.003	3115.468
139.4841	505.139	936.7251	1244.386	1517.034	3117.29
146.3005	532.9971	942.8718	1255.672	1521.952	3119.06
154.162	533.2995	945.4902	1272.509	1523.141	3123.735
167.0263	549.4314	958.3199	1297.767	1524.172	3125.19
174.7787	553.4745	965.5062	1305.898	1526.131	3127.709
178.6579	574.5121	988.4841	1311.936	1526.63	3127.8
186.1298	580.0112	990.0287	1320.99	1528.017	3145.673
188.1012	585.0303	992.043	1329.348	1528.626	3147.737
197.2169	615.1055	1011.986	1330.147	1538.452	3151.949
202.6146	634.9786	1026.005	1341.853	1541.901	3157.723
205.4852	651.3553	1028.619	1346.977	1568.512	3175.448
213.1737	667.9582	1031.859	1347.281	1583.424	3177.697
223.1641	674.0627	1034.189	1351.739	1598.746	3183.358
231.2493	683.8062	1040.051	1361.592	1602.477	3196.672

241.258	696.168	1051.917	1373.282	1609.28	3201.633
244.0792	703.0604	1066.878	1378.602	1629.303	3208.725
247.8784	715.873	1067.514	1388.116	1654.283	3241.756
252.7377	720.9933	1068.319	1390.616	1700.415	3257.544

Table S6. Calculated frequencies (cm^{-1}) of excited state chlorophyll a using B3LYP functional with 6-31G (d) basis set.

10.7602	261.8641	717.5331	1066.288	1386.711	1730.197
11.659	271.5634	724.3005	1067.9	1398.757	1785.772
20.7234	276.6938	725.9355	1075.633	1401.963	1804.928
24.3337	286.4552	731.3397	1078.633	1418.798	1844.21
34.0678	295.9137	742.64	1087.671	1423.369	3027.087
42.6836	301.3887	752.0364	1095.179	1434.619	3029.428
43.2155	305.0981	756.5658	1098.389	1436.033	3036.765
47.9279	318.3835	765.7633	1106.881	1436.456	3036.976
51.6617	321.4842	776.2877	1132.237	1441.564	3043.375
59.1931	343.601	777.3691	1133.627	1443.72	3045.762
61.435	347.6679	786.6949	1145	1445.174	3048.416
64.3318	355.8395	795.4906	1146.851	1451.302	3051.114
72.1561	373.3218	800.2848	1149.904	1460.656	3062.708
77.5545	379.7403	808.2336	1154.967	1473.427	3064.626
83.9433	383.647	829.3095	1166.965	1478.685	3067.046
91.275	395.8375	838.6249	1176.041	1485.49	3072.875
95.5947	410.8077	841.7152	1182.178	1491.526	3073.392
97.2561	415.6543	843.0785	1184.802	1495.127	3076.31
111.035	425.4949	851.614	1196.229	1495.842	3078.443
121.3568	454.1778	877.9749	1199.856	1508.213	3081.557
122.9871	463.6895	905.4192	1204.397	1512.163	3082.937
123.6632	471.9574	911.7861	1212.762	1512.424	3087.568
128.6014	473.3499	912.3807	1220.404	1513.061	3108.894
131.5825	482.2358	927.9818	1226.901	1513.988	3116.975
138.633	496.6277	933.3644	1242.347	1516.368	3119.143
145.1648	526.7973	938.1909	1252.986	1517.034	3120.158
153.3472	529.4973	944.4231	1269.039	1522.553	3120.488
165.577	537.743	959.0006	1275.181	1522.862	3126.395
170.7407	551.0973	961.9917	1296.079	1523.744	3130.372
178.1012	574.7096	985.7357	1307.714	1525.711	3130.864
185.8073	576.4033	989.7283	1309.07	1526.235	3144.442
190.3726	578.7644	992.0477	1319.884	1527.39	3147.38
197.2684	608.2724	1009.903	1328.848	1529.23	3147.692
201.2104	630.6347	1023.168	1333.984	1539.202	3158.119
204.2641	645.1427	1025.084	1338.653	1539.941	3175.837
210.3243	664.565	1027.904	1343.195	1561.757	3179.569
222.5162	666.2892	1029.127	1348.38	1571.383	3183.595
228.2014	682.456	1038.412	1350.52	1577.555	3199.258

235.6452	689.8434	1040.689	1360.207	1597.196	3210.707
243.2033	698.194	1062.557	1370.24	1626.407	3215.084
247.0451	708.0714	1063.728	1374.815	1639.7	3242.417
247.8962	715.8251	1065.741	1384.782	1681.869	3260.793

Table S7. Calculated frequencies (cm^{-1}) of ground state chlorophyll f using B3LYP functional with 6-31G (d) basis set.

10.4591	262.3759	726.7941	1068.04	1402.655	1802.544
14.872	263.6149	734.8067	1068.632	1414.494	1813.679
20.8463	278.0363	736.8524	1068.91	1419.19	1843.731
24.5097	287.021	743.5688	1078.626	1421.964	2927.211
34.6378	292.2455	749.6178	1089.104	1427.845	3039.308
39.534	304.4148	755.0199	1092.953	1434.302	3040.544
41.0606	310.9425	759.2409	1114.038	1437.36	3041.944
43.0419	319.149	771.4307	1135.268	1437.99	3046.162
49.283	326.0078	776.739	1137.266	1442.109	3048.427
54.9475	343.2559	783.2142	1148.275	1447.036	3050.69
59.3939	350.3829	786.5754	1155.624	1462.429	3054.856
63.6328	353.6995	798.5174	1161.713	1467.802	3062.293
68.8011	373.864	804.2461	1177.319	1476.139	3067.473
70.9448	378.4298	808.0676	1178.538	1478.441	3068.03
77.2025	391.8527	833.3289	1182.762	1486.982	3074.663
87.9743	397.243	845.7578	1185.547	1491.503	3074.997
95.4884	401.1977	857.6897	1191.1	1494.867	3078.521
104.5617	412.728	883.1526	1196.831	1495.25	3080.029
114.1464	414.9086	886.0092	1197.504	1509.272	3087.187
118.7878	439.0238	902.6189	1209.916	1513.479	3089.817
120.9649	453.6281	913.8517	1212.193	1515.166	3110.089
123.0354	473.1728	917.116	1220.013	1515.335	3115.975
127.4572	480.6497	919.116	1231.32	1516.36	3117.712
132.3791	494.3623	933.295	1231.776	1516.775	3120
135.6637	495.7926	942.2825	1247.801	1523.09	3125.093
141.5309	516.9247	943.3225	1261.291	1524.011	3130.029
148.2326	532.191	951.0137	1276.081	1526.156	3131.083
157.2183	544.6504	954.0021	1298.525	1526.367	3147.605
167.8946	552.5605	958.6542	1308.878	1527.683	3149.485
173.9284	570.7764	973.2881	1314.571	1528.001	3157.767
179.7438	575.1739	988.8084	1321.172	1537.33	3160.832
187.1529	585.1635	989.561	1329.189	1541.402	3175.01
190.752	587.3458	993.3135	1333.931	1549.841	3175.914
200.3268	622.8327	1012.801	1341.425	1563.884	3185.016
202.9293	639.9768	1017.064	1343.009	1588.298	3205.206
210.5623	644.7205	1027.733	1348.02	1598.509	3224.028
212.3942	674.5273	1028.379	1351.231	1613.48	3228.227
229.56	676.6134	1032.219	1362.385	1629.979	3241.628

230.2841	689.3317	1039.529	1376.091	1653.057	3257.422
239.7744	698.5601	1048.351	1383.587	1698.601	
244.2846	705.9693	1050.629	1390.53	1730.257	
245.4764	716.1972	1066.78	1398.963	1759.315	

Table S8. Calculated frequencies (cm^{-1}) of excited state chlorophyll f using B3LYP functional with 6-31G (d) basis set.

10.2963	256.4439	720.5583	1064.677	1390.226	1790.343
14.8506	262.1067	727.0363	1065.592	1401.877	1806.868
21.0533	272.2852	729.7905	1068.125	1407.212	1843.282
24.338	285.4985	739.3153	1073.779	1417.748	2931.506
34.5328	289.0486	741.1066	1079.135	1420.098	3032.922
39.493	304.1183	746.3481	1088.123	1428.589	3037.498
41.824	308.8558	752.8279	1098.471	1435.625	3040.154
43.132	317.3182	765.0566	1109.639	1436.779	3045.349
51.0771	320.4958	772.3414	1125.099	1438.32	3050.824
55.8931	341.3302	777.4681	1134.196	1443.674	3051.348
62.1183	348.3064	785.9144	1141.302	1446.798	3051.827
66.1299	352.5445	794.6005	1148.644	1453.275	3063.514
71.6067	370.2035	802.4907	1151.548	1456.904	3066.42
76.4133	377.0179	807.4981	1165.745	1470.71	3068.391
79.7064	381.3502	823.9891	1171.42	1478.367	3074.75
89.0443	388.9468	843.6341	1180.507	1480.585	3075.737
94.6936	401.3515	849.7431	1184.633	1489.666	3077.725
107.8766	410.8326	853.4123	1187.509	1492.972	3080.361
116.7737	414.9151	870.6237	1196.61	1495.058	3081.248
121.7158	432.4023	885.9698	1202.093	1509.074	3084.359
123.0176	448.6444	901.857	1203.859	1512.596	3110.495
128.8587	470.0457	903.9873	1211.283	1512.808	3119.862
129.3667	472.4392	914.0227	1217.693	1513.211	3120.365
132.5299	476.3848	927.5612	1228.166	1516.029	3121.672
134.9233	488.5153	930.6865	1237.655	1517.002	3126.467
140.0078	506.3061	933.3935	1259.805	1522.731	3132.359
147.7273	528.9308	941.3063	1265.616	1523.762	3135.054
161.337	534.7007	948.8163	1277.601	1525.929	3146.232
167.372	552.0746	958.9711	1297.873	1526.423	3149.506
172.2993	569.2409	968.9846	1310.296	1527.637	3156.807
178.4006	575.0682	986.9972	1311.481	1527.782	3157.957
185.4195	576.5575	989.0976	1320.573	1534.015	3175.355
191.1003	583.0433	993.2882	1328.738	1540.543	3177.743
198.4879	618.6264	1003.746	1336.694	1545.953	3185.383
201.5721	637.8746	1008.192	1340.956	1562.397	3211.542
208.5516	640.9052	1015.128	1343.336	1577.558	3229.775
210.2801	669.0326	1022.555	1347.081	1599.448	3236.257
228.5269	671.2643	1028.709	1349.73	1625.803	3242.09

229.0105	686.7814	1030.633	1361.809	1638.126	3259.703
235.8247	693.1521	1039.662	1372.418	1677.077	
240.9382	697.4235	1048.039	1376.41	1730.061	
246.4869	709.8973	1063.185	1384.579	1735.914	

Table S9. Dimensionless displacements (δ) of chlorophyll a normal modes (n) calculated using CAM-B3LYP functional with 6-31G (d) basis set.

n	δ	n	δ	n	δ	n	δ	n	δ	n	δ
1	33.4746	43	-3.15461	85	0.095745	127	-0.70819	169	-0.11142	211	-0.01426
2	13.9592	44	1.4687	86	-0.94924	128	0.818405	170	0.499479	212	0.375928
3	41.6757	45	0.263922	87	-0.6355	129	-0.78926	171	0.094264	213	0.252301
4	-16.962	46	-0.2658	88	-0.1343	130	-0.11105	172	0.207575	214	0.025171
5	-10.3347	47	-0.07958	89	-3.40713	131	0.444346	173	-0.2502	215	1.52912
6	41.346	48	2.78124	90	1.46628	132	0.192033	174	-0.00598	216	-0.15074
7	-36.9205	49	-1.26858	91	-1.5324	133	-0.25378	175	-0.87951	217	-0.01077
8	2.12973	50	-1.223	92	0.271639	134	-0.52181	176	-0.12997	218	-0.2237
9	-15.6558	51	-0.73201	93	0.819387	135	0.640049	177	-0.38205	219	1.10567
10	18.7425	52	-0.17944	94	2.15929	136	1.04908	178	0.881713	220	0.444904
11	15.3836	53	-1.99073	95	0.944051	137	0.085335	179	-0.00417	221	-0.41838
12	-31.5036	54	1.53594	96	-0.25827	138	0.527816	180	0.7267	222	0.009897
13	3.19918	55	2.77102	97	0.195637	139	-1.64239	181	0.082995	223	-0.06515
14	-2.77557	56	-0.91955	98	-0.51353	140	0.309776	182	0.037925	224	-0.01955
15	-11.7955	57	-1.22602	99	0.26925	141	-0.78301	183	0.461108	225	-0.05266
16	-18.8608	58	2.55739	100	0.572524	142	-1.0029	184	0.760799	226	-0.00112
17	-5.66861	59	-1.32455	101	-0.08244	143	2.18004	185	0.679826	227	0.003483
18	4.74174	60	0.291322	102	-0.54048	144	-0.40257	186	0.003127	228	-0.02953
19	-6.3398	61	0.576431	103	-0.21495	145	-0.31457	187	-0.34555	229	-0.00289
20	0.190271	62	0.683232	104	1.22909	146	1.42064	188	-0.49159	230	-0.0116
21	-0.644859	63	1.17248	105	-0.54323	147	0.140233	189	0.155533	231	0.40682
22	-2.28174	64	2.23727	106	2.65731	148	0.929742	190	0.163172	232	-0.02354
23	-3.70158	65	1.82166	107	1.16072	149	-0.52868	191	0.138094	233	-0.02956
24	-0.643825	66	-0.77868	108	-0.06466	150	-0.80667	192	-0.11119	234	-0.13558
25	4.01129	67	1.83242	109	0.039761	151	0.930259	193	-0.78359	235	-0.01852
26	7.79859	68	-1.91807	110	-1.48823	152	-3.74361	194	-0.19772	236	0.006701
27	6.50439	69	-1.59473	111	0.003611	153	-1.9575	195	-0.10492	237	0.014517
28	-4.1157	70	-0.53419	112	-1.42196	154	-0.2992	196	0.156308	238	-0.01525
29	1.54834	71	-1.47952	113	0.214032	155	1.50726	197	0.155833	239	0.05461
30	3.78521	72	0.516475	114	0.39151	156	-0.54209	198	-0.48451	240	-0.19377
31	-2.70305	73	1.46983	115	-0.0074	157	-1.15506	199	0.459945	241	0.204968
32	3.76605	74	-0.82265	116	-0.26863	158	1.03417	200	-0.07341	242	0.008734
33	-1.87025	75	1.26389	117	-4.60375	159	-0.38169	201	-0.90415	243	0.008445
34	-1.65588	76	-0.10794	118	0.752536	160	0.070782	202	2.51592	244	-0.27399
35	2.01914	77	1.08422	119	-0.11884	161	-0.01395	203	-0.42523	245	-0.59167
36	-0.635426	78	-0.36782	120	0.055994	162	-0.67223	204	2.11249	246	-0.00815
37	4.34028	79	0.332361	121	0.078874	163	-0.66249	205	0.46036	247	0.012327
38	-1.55607	80	0.34084	122	-0.10897	164	0.98235	206	1.08319	248	0.117622
39	-1.19897	81	-1.48731	123	-1.74314	165	-4.47469	207	0.337611	249	-0.07171
40	-2.65367	82	0.482347	124	-0.01851	166	-0.49652	208	1.45246	250	-0.13034
41	-1.67822	83	0.439125	125	0.058768	167	1.01484	209	-0.8564	251	0.003019
42	1.88576	84	-0.91704	126	-0.29524	168	0.262865	210	0.909355	252	0.379305

Table S10. Dimensionless displacements (δ) of chlorophyll a normal modes (n) calculated using B3LYP functional with 6-31G (d) basis set.

n	δ	n	δ	n	δ	n	δ	n	δ	n	δ
1	4.35058	4	-	85	0.30157	12	0.04932	16	-	21	-
2	-37.588	4	-	86	-	12	-	17	-	21	0.46410
3	39.6875	4	0.55962	87	-	12	-	17	-0.1078	21	0.37818
4	-	4	-	88	0.18459	13	0.42039	17	0.70216	21	0.06833
5	16.7216	4	0.68843	89	0.47707	13	-	17	0.37410	21	0.83799
6	-	4	2.55961	90	-	13	-	17	-	21	-
7	11.2846	4	1.28566	91	-	13	0.38182	17	-	21	-
8	-	5	0.06662	92	0.44677	13	0.21366	17	-	21	0.09634
9	-	5	1.77021	93	0.26754	13	-	17	0.10527	21	0.08609
1	-	5	-	94	-	13	-	17	-	22	0.00796
1	16.6519	5	-	95	1.62442	13	0.5635	17	-	22	0.04936
1	-	5	-	96	-	13	-	18	-	22	0.07046
1	11.1514	5	2.9064	97	0.32982	13	0.25358	18	-	22	0.03189
1	1.17269	5	1.14593	98	-	14	-	18	-	22	0.00283
1	-	5	0.77068	99	0.60503	14	-	18	0.85975	22	0.01985
1	-	5	-1.9577	10	0.06471	14	0.31978	18	-	22	-
1	9.89301	5	0.20537	10	-	14	0.76859	18	0.03969	22	-
1	-	6	0.19055	10	-	14	0.29005	18	0.21197	22	-
1	2.3075	6	0.05088	10	0.96809	14	0.70516	18	0.77848	22	-
2	0.64627	6	0.33217	10	0.06061	14	0.76915	18	-	23	0.00023
2	0.47549	6	0.24192	10	0.10605	14	0.78063	18	-	23	-
2	-	6	1.74938	10	-	14	1.43051	19	-	23	0.31672
2	-	6	-	10	-	14	0.06577	19	0.04103	23	0.01037
2	0.40212	6	-	10	0.44947	15	-	19	0.04263	23	-
2	2.62326	6	0.45151	10	0.81312	15	-	19	0.01598	23	0.01903
2	-	6	0.25071	11	0.70524	15	-	19	0.18629	23	-
2	-	6	1.76894	11	0.61374	15	-	19	0.00527	23	-
2	-	7	0.54828	11	-0.0087	15	0.33278	19	0.09795	23	0.00345
2	1.59253	7	-	11	0.35857	15	0.53327	19	-	23	0.03889
3	-	7	0.33840	11	-	15	-	19	-	24	-0.0048
3	-2.6011	7	1.47623	11	0.03668	15	-	19	-	24	0.0309
3	1.90577	7	-	11	-	15	-	20	-	24	0.00723
3	-	7	0.61601	11	2.53948	15	-	20	-	24	-
3	-	7	0.08141	11	0.08160	16	0.26388	20	0.62884	24	0.02773
3	3.78946	7	0.57892	11	0.10777	16	-	20	0.49495	24	-
3	1.4176	7	-1.0968	12	-	16	-	20	-	24	-
3	1.97536	7	-	12	-	16	-	20	-	24	0.00557
3	0.54238	8	-	12	0.07158	16	-	20	0.61635	24	-
3	0.01622	8	0.80643	12	-0.4725	16	2.52879	20	-	24	0.07629
4	-	8	-	12	0.02732	16	-	20	-	25	-
4	-	8	-	12	-	16	-	20	0.38563	25	0.0133
4	2.61936	8	-	12	0.00684	16	0.23311	21	0.25473	25	0.28322

Table S11. Cartesian coordinates of ground state chlorophyll a (CAM-B3LYP functional with 6-31G (d) basis set)

C	-1.66352	-3.84832	-0.09832
C	-0.24333	-3.54237	-0.01687
C	-0.08212	-2.13826	-0.05982
C	-1.29543	-1.41182	-0.15022
C	-2.40942	-2.4496	-0.13166
C	1.02613	-4.09512	0.08341
C	1.91745	-2.96292	0.09829
N	1.1921	-1.77952	0.00587
C	-1.37371	-0.05097	-0.23579
N	-0.25277	0.75465	-0.25947
C	-0.60985	2.03688	-0.46349
C	-2.10398	2.16568	-0.71731
C	-2.64075	0.77864	-0.30397
C	0.23744	3.13689	-0.49098
C	1.63438	3.15335	-0.34812
C	2.46194	4.35353	-0.36928
C	3.75591	3.93533	-0.22403
C	3.7192	2.47902	-0.10254
N	2.40779	2.05752	-0.18431
C	4.98033	4.73239	-0.16078
C	5.22016	5.85621	-0.84247
C	1.9384	5.74894	-0.48281
Mg	1.78018	0.13855	-0.08215
N	3.72366	-0.54166	0.09486
C	4.82792	0.23787	0.13209
C	6.01362	-0.57065	0.26814
C	5.59054	-1.87745	0.30791
C	4.15022	-1.84743	0.19886
C	4.81081	1.65367	0.03826
C	-2.39299	2.50804	-2.18356
C	-3.38327	0.79229	1.04288
C	-4.75264	1.45762	0.94087
C	-5.45922	1.5159	2.28082
O	-4.95675	1.14686	3.31124
C	7.41414	-0.04621	0.35087
C	3.30884	-2.96318	0.1923
C	6.43155	-3.10864	0.47486
C	6.608	-3.52563	1.94132
C	1.40456	-5.53917	0.14895
C	-3.34189	-2.41818	-1.32176
O	-3.04044	-2.06781	-2.44034
O	-2.24045	-4.91426	-0.13304
O	-4.56284	-2.8589	-0.99071
C	-5.48769	-3.01029	-2.07166
O	-6.70763	2.04176	2.32802
C	-7.45572	2.3802	1.15263

C	-8.15069	1.18632	0.57016
H	-0.2409	4.09717	-0.65336
H	5.78159	2.13745	0.06861
H	-3.30637	0.3763	-1.07722
H	3.79044	-3.93382	0.26973
H	-2.51756	2.95718	-0.08137
H	5.75979	4.35752	0.50066
H	-3.01973	-2.41086	0.77687
H	-1.99995	1.73108	-2.84746
H	-3.47254	2.58279	-2.35506
H	-1.93585	3.46052	-2.46792
H	-5.6699	-2.04838	-2.55639
H	-6.40118	-3.39552	-1.62086
H	-5.09441	-3.71541	-2.8068
H	0.99291	5.86362	0.05608
H	1.75548	6.0347	-1.52644
H	2.65102	6.46613	-0.06681
H	-3.51637	-0.22862	1.411
H	-2.77488	1.30502	1.79521
H	-5.38445	0.91906	0.22533
H	-4.66081	2.48208	0.55685
H	2.06575	-5.74469	0.99785
H	0.51382	-6.1629	0.24978
H	1.93185	-5.85267	-0.75974
H	-6.82861	2.88222	0.40987
H	-8.18942	3.10482	1.51589
H	7.57729	0.53398	1.26711
H	8.14339	-0.86053	0.34922
H	7.65329	0.60817	-0.4952
H	7.4179	-2.93875	0.02915
H	5.99243	-3.94014	-0.08804
H	7.22467	-4.4275	2.01965
H	7.09224	-2.73071	2.51765
H	5.64111	-3.73166	2.4116
H	6.15831	6.3898	-0.72546
H	4.50873	6.26647	-1.55236
C	-8.16916	0.89367	-0.72705
H	-8.68446	0.56413	1.28649
H	-7.64158	1.50182	-1.45922
H	-8.72265	0.04179	-1.10988

Table S12. Cartesian coordinates of excited state chlorophyll a (CAM-B3LYP functional with 6-31G (d) basis set)

C	-1.66995	-3.85435	-0.07817
C	-0.25035	-3.54912	0.0002

C	-0.09067	-2.13819	-0.05424
C	-1.30106	-1.42099	-0.14946
C	-2.41507	-2.45551	-0.12386
C	1.01646	-4.09772	0.10406
C	1.91404	-2.95545	0.10919
N	1.19111	-1.7762	0.00846
C	-1.37141	-0.05047	-0.24848
N	-0.24906	0.7455	-0.28006
C	-0.61498	2.03803	-0.4998
C	-2.10402	2.1523	-0.76976
C	-2.63656	0.77534	-0.32243
C	0.22677	3.13872	-0.52478
C	1.62961	3.17282	-0.372
C	2.4467	4.35816	-0.3864
C	3.75313	3.93422	-0.23056
C	3.70766	2.4873	-0.11458
N	2.41314	2.06251	-0.20413
C	4.97708	4.72088	-0.15676
C	5.19759	5.91722	-0.7165
C	1.93704	5.75908	-0.49831
Mg	1.77832	0.14521	-0.09283
N	3.71483	-0.54215	0.09743
C	4.83011	0.25293	0.12661
C	6.02436	-0.57591	0.26301
C	5.60056	-1.86985	0.30997
C	4.14248	-1.83647	0.20409
C	4.82276	1.64056	0.03216

C	-2.38745	2.44647	-2.24916
C	-3.36359	0.81627	1.03403
C	-4.73463	1.47894	0.93489
C	-5.42615	1.55459	2.282
O	-4.91095	1.19851	3.31071
C	7.42007	-0.04553	0.33498
C	3.31037	-2.96665	0.20331
C	6.42224	-3.11308	0.47159
C	6.4986	-3.6043	1.92405
C	1.41238	-5.53313	0.19311
C	-3.34516	-2.43234	-1.31625
O	-3.04869	-2.06276	-2.43029
O	-2.24582	-4.92264	-0.1039
O	-4.5562	-2.9031	-0.99272
C	-5.47491	-3.06677	-2.07724
O	-6.6741	2.07956	2.3369
C	-7.43555	2.40467	1.16619
C	-8.13331	1.20353	0.60236
H	0.25779	4.09398	-0.69835
H	5.79238	2.12495	0.06101
H	-3.30982	0.35271	-1.07793
H	3.79244	-3.93507	0.28392
H	-2.52762	2.96122	-0.16296
H	5.79152	4.27579	0.41299
H	-3.02782	-2.40974	0.78311
H	-1.98732	1.65052	-2.88569
H	-3.46671	2.51037	-2.42752

H	-1.93339	3.39171	-2.56061
H	-5.67797	-2.1054	-2.55471
H	-6.3803	-3.47648	-1.63178
H	-5.06388	-3.75689	-2.81686
H	0.92377	5.85036	-0.09626
H	1.90803	6.10833	-1.53885
H	2.57795	6.45033	0.05744
H	-3.49157	-0.19675	1.42484
H	-2.746	1.34531	1.76725
H	-5.37366	0.92996	0.23386
H	-4.64677	2.49776	0.5355
H	1.9582	-5.74156	1.12155
H	0.53026	-6.17632	0.16553
H	2.06879	-5.8191	-0.63797
H	-6.81762	2.90169	0.41244
H	-8.16728	3.13067	1.53059
H	7.56499	0.58143	1.22282
H	8.15289	-0.85473	0.38175
H	7.66092	0.5704	-0.53914
H	7.43637	-2.92983	0.10096
H	6.01386	-3.91043	-0.16074
H	7.09466	-4.52036	1.99426
H	6.95963	-2.84751	2.5668
H	5.50224	-3.81539	2.32513
H	6.14893	6.42339	-0.58541
H	4.46058	6.41694	-1.33612
C	-8.16533	0.89892	-0.69183

H	-8.65752	0.5866	1.33021
H	-7.64769	1.50178	-1.4354
H	-8.7207	0.0421	-1.06059

Table S13. Cartesian coordinates of ground state chlorophyll f (CAM-B3LYP functional with 6-31G (d) basis set)

C	-1.68218	-3.94285	0.00138
C	-0.25697	-3.63037	0.05728
C	-0.10563	-2.21859	-0.02069
C	-1.3185	-1.51144	-0.11362
C	-2.43115	-2.55051	-0.0652
C	1.00587	-4.17366	0.14568
C	1.903	-3.02771	0.11913
N	1.16818	-1.85176	0.01559
C	-1.4013	-0.14336	-0.23616
N	-0.29408	0.65684	-0.29184
C	-0.6619	1.94579	-0.51991
C	-2.15939	2.05496	-0.75657
C	-2.67771	0.67085	-0.31229
C	0.16968	3.04277	-0.57989
C	1.57521	3.07391	-0.43742
C	2.40018	4.26469	-0.4742
C	3.71015	3.85851	-0.27875
C	3.67447	2.42089	-0.1617
N	2.3617	1.99443	-0.24623
C	4.89797	4.71568	-0.21735
C	5.87467	4.61268	0.68765

C	1.96932	5.64704	-0.63576
Mg	1.74172	0.06928	-0.11806
N	3.6915	-0.60699	0.04455
C	4.8024	0.18438	0.03486
C	5.99128	-0.62367	0.13437
C	5.56925	-1.92986	0.20455
C	4.12683	-1.89744	0.14574
C	4.77777	1.58561	-0.05851
C	-2.47323	2.3719	-2.22321
C	-3.40828	0.69633	1.04099
C	-4.77272	1.37348	0.95109
C	-5.48351	1.39196	2.29009
O	-4.99596	0.96051	3.30352
C	7.39447	-0.10141	0.16162
C	3.28435	-3.02878	0.17867
C	6.41374	-3.16149	0.3523
C	6.63033	-3.57575	1.81405
C	1.39802	-5.61065	0.24375
C	-3.35947	-2.55406	-1.26126
O	-3.04605	-2.2452	-2.38841
O	-2.24859	-5.01325	0.0027
O	-4.58465	-2.9727	-0.92176
C	-5.50869	-3.14635	-2.00118
O	-6.71775	1.94649	2.35703
C	-7.4486	2.36643	1.197
C	-8.17007	1.22394	0.54756
H	-0.29582	4.0047	-0.75813

H	5.7439	2.07788	-0.06796
H	-3.34412	0.24427	-1.07193
H	3.7726	-3.99546	0.25725
H	-2.56997	2.85344	-0.12859
H	4.96087	5.50888	-0.96
H	-3.04512	-2.48973	0.83958
H	-2.07786	1.59196	-2.88255
H	-3.55615	2.42887	-2.38051
H	-2.03383	3.32729	-2.5238
H	-5.67655	-2.19767	-2.5159
H	-6.42821	-3.50534	-1.54135
H	-5.12187	-3.87914	-2.7122
H	2.78013	6.39905	-0.57926
H	-3.54746	-0.32103	1.41605
H	-2.78864	1.20815	1.78489
H	-5.40709	0.86082	0.21858
H	-4.67143	2.40731	0.59739
H	1.96318	-5.80739	1.16181
H	0.51244	-6.24931	0.24461
H	2.032	-5.90733	-0.59945
H	-6.80373	2.89127	0.48608
H	-8.16591	3.08941	1.59451
H	7.59421	0.47675	1.07177
H	8.12158	-0.91683	0.13
H	7.60012	0.55408	-0.69186
H	7.38753	-2.99343	-0.12066
H	5.95972	-3.99408	-0.19728

H	7.24788	-4.47812	1.87736
H	7.13145	-2.78018	2.37483
H	5.67712	-3.77973	2.31256
H	6.72185	5.29131	0.6722
H	5.84744	3.87593	1.48498
C	-8.18747	1.00192	-0.76364
H	-8.72548	0.57908	1.22647
H	-7.63922	1.63435	-1.4591
H	-8.7613	0.18739	-1.19483
O	0.82313	6.02029	-0.82134

Table S14. Cartesian coordinates of excited state chlorophyll f (CAM-B3LYP functional with 6-31G (d) basis set)

C	-1.67077	-3.95125	-0.00868
C	-0.25174	-3.63429	0.05092
C	-0.10408	-2.22362	-0.01585
C	-1.32032	-1.5167	-0.10555
C	-2.42672	-2.55959	-0.05912
C	1.02043	-4.17275	0.13778
C	1.9081	-3.0236	0.12016
N	1.17655	-1.85152	0.02533
C	-1.40357	-0.14862	-0.22444
N	-0.28512	0.65524	-0.27808
C	-0.66058	1.93834	-0.51406
C	-2.15363	2.04812	-0.75177
C	-2.67448	0.66671	-0.30556
C	0.16992	3.04949	-0.57559
C	1.56816	3.08969	-0.4324
C	2.38536	4.28109	-0.46535
C	3.70164	3.87755	-0.25893
C	3.6676	2.43532	-0.14654
N	2.3698	2.00136	-0.23755
C	4.87426	4.74462	-0.19854
C	5.93008	4.59377	0.61008
C	1.9342	5.6529	-0.62629
Mg	1.7467	0.07309	-0.1038

N	3.69141	-0.59753	0.05742
C	4.80115	0.20049	0.04371
C	6.00591	-0.62129	0.13634
C	5.59155	-1.9165	0.20557
C	4.13029	-1.89205	0.15259
C	4.78913	1.59011	-0.04272
C	-2.46804	2.36487	-2.22015
C	-3.41272	0.69911	1.04511
C	-4.77577	1.3776	0.94385
C	-5.49197	1.40441	2.28008
O	-5.00665	0.98072	3.29783
C	7.39922	-0.08085	0.15406
C	3.30772	-3.02413	0.18091
C	6.42622	-3.15395	0.34331
C	6.56174	-3.63361	1.79529
C	1.42811	-5.60496	0.22758
C	-3.36662	-2.553	-1.2446
O	-3.07688	-2.19641	-2.36456
O	-2.23693	-5.0241	-0.01902
O	-4.57332	-3.02163	-0.90627
C	-5.5023	-3.19645	-1.98105
O	-6.72678	1.95745	2.33844
C	-7.45434	2.36744	1.17255
C	-8.16939	1.21834	0.52776
H	-0.3028	4.00746	-0.75503
H	5.7549	2.07916	-0.06077
H	-3.33793	0.23655	-1.0656
H	3.79795	-3.98924	0.25202
H	-2.56021	2.85117	-0.12636
H	4.86015	5.61006	-0.85874
H	-3.03082	-2.5087	0.85306
H	-2.08006	1.58021	-2.87801
H	-3.55099	2.42832	-2.37302
H	-2.02386	3.31693	-2.52353
H	-5.70922	-2.23986	-2.46615
H	-6.40351	-3.60099	-1.52281
H	-5.09782	-3.89439	-2.71686
H	2.72821	6.42109	-0.55945
H	-3.55477	-0.31676	1.42281
H	-2.79625	1.21334	1.78989
H	-5.40715	0.8616	0.21123
H	-4.67146	2.40951	0.58518
H	1.99045	-5.80469	1.14786

H	0.55016	-6.25425	0.21808
H	2.07201	-5.89068	-0.61315
H	-6.80809	2.88978	0.46099
H	-8.17536	3.09075	1.56257
H	7.57518	0.54448	1.0375
H	8.13912	-0.88471	0.16892
H	7.60023	0.54003	-0.72652
H	7.42384	-2.96696	-0.06777
H	5.99862	-3.95799	-0.26716
H	7.16691	-4.54467	1.84854
H	7.04168	-2.86912	2.41458
H	5.58327	-3.84929	2.236
H	6.75182	5.30261	0.58561
H	5.99464	3.79295	1.34012
C	-8.18049	0.98756	-0.782
H	-8.72572	0.57631	1.20858
H	-7.63157	1.61725	-1.47943
H	-8.74991	0.16837	-1.21016
O	0.78057	6.00683	-0.82243

Table S15. Cartesian coordinates of ground state chlorophyll a (B3LYP functional with 6-31G (d) basis set)

C	-1.64853	-3.88394	-0.07638
C	-0.23019	-3.55753	-0.00367
C	-0.07897	-2.14594	-0.0551
C	-1.29374	-1.42924	-0.14696
C	-2.40838	-2.4733	-0.1289
C	1.04473	-4.10459	0.09847
C	1.93746	-2.96204	0.10467
N	1.2043	-1.7804	0.00635
C	-1.38095	-0.05396	-0.23653
N	-0.26596	0.75225	-0.26711
C	-0.62487	2.04522	-0.47224
C	-2.12543	2.1686	-0.72347

C	-2.6579	0.77182	-0.30588
C	0.22586	3.14411	-0.50111
C	1.62901	3.17803	-0.36094
C	2.44807	4.37665	-0.38194
C	3.75816	3.9607	-0.2344
C	3.72291	2.50538	-0.11554
N	2.41471	2.07603	-0.19751
C	4.97846	4.75786	-0.16235
C	5.2039	5.94246	-0.75115
C	1.92694	5.77717	-0.49082
Mg	1.7895	0.14749	-0.0907
N	3.74084	-0.53078	0.09004
C	4.85087	0.26275	0.12104
C	6.04388	-0.55014	0.25699
C	5.62221	-1.86129	0.30388
C	4.17363	-1.83494	0.19832
C	4.82708	1.6708	0.02466
C	-2.42384	2.51282	-2.19397
C	-3.40777	0.78104	1.04559
C	-4.78238	1.45083	0.95247
C	-5.49604	1.47598	2.29745
O	-5.00716	1.05483	3.31766
C	7.44553	-0.02034	0.33235
C	3.33333	-2.95968	0.19628
C	6.46159	-3.09731	0.4754
C	6.56313	-3.56981	1.93887
C	1.4233	-5.55061	0.18342

C	-3.34325	-2.44352	-1.32522
O	-3.10999	-1.92396	-2.39734
O	-2.21246	-4.95975	-0.0921
O	-4.48521	-3.10204	-1.0457
C	-5.40226	-3.24215	-2.1417
O	-6.74082	2.03136	2.3634
C	-7.47333	2.44568	1.19342
C	-8.18703	1.29646	0.53966
H	-0.25738	4.10227	-0.6667
H	5.79601	2.15907	0.05014
H	-3.31844	0.36186	-1.07914
H	3.81806	-3.92847	0.27595
H	-2.5436	2.95905	-0.08729
H	5.78518	4.32919	0.43188
H	-3.0261	-2.4359	0.77684
H	-2.03656	1.73435	-2.86068
H	-3.50484	2.59149	-2.36019
H	-1.96424	3.46483	-2.48032
H	-5.72799	-2.26131	-2.49967
H	-6.24624	-3.80678	-1.74482
H	-4.9326	-3.78371	-2.96681
H	0.93877	5.87405	-0.02852
H	1.82931	6.10445	-1.5355
H	2.60086	6.48228	0.00654
H	-3.54227	-0.2426	1.40903
H	-2.80025	1.28864	1.8035
H	-5.41408	0.93138	0.22055

H	-4.68892	2.48393	0.58908
H	1.96352	-5.77278	1.11269
H	0.52878	-6.17764	0.15152
H	2.07707	-5.84583	-0.64725
H	-6.83102	2.97486	0.48138
H	-8.19699	3.16662	1.589
H	7.61883	0.54367	1.2586
H	8.18163	-0.82915	0.30251
H	7.66849	0.65661	-0.50191
H	7.47116	-2.91117	0.08956
H	6.05524	-3.91147	-0.13843
H	7.17513	-4.47609	2.01509
H	7.01781	-2.79701	2.56826
H	5.5726	-3.79081	2.35118
H	6.15059	6.45819	-0.61878
H	4.47652	6.42242	-1.39798
C	-8.19358	1.06515	-0.77344
H	-8.74512	0.65255	1.21827
H	-7.64533	1.69632	-1.47055
H	-8.75901	0.24449	-1.20578

Table S16. Cartesian coordinates of excited state chlorophyll a (B3LYP functional with 6-31G (d) basis set)

C	-1.65876	-3.89063	-0.06134
C	-0.24308	-3.566	0.00793
C	-0.08856	-2.15143	-0.04778
C	-1.30339	-1.43577	-0.14222

C	-2.41715	-2.47719	-0.11911
C	1.03307	-4.11177	0.11389
C	1.92976	-2.96387	0.11619
N	1.19938	-1.78303	0.01406
C	-1.38067	-0.05575	-0.24139
N	-0.2567	0.74432	-0.27846
C	-0.62371	2.04138	-0.50322
C	-2.1186	2.15529	-0.76737
C	-2.6515	0.77095	-0.31649
C	0.22153	3.14596	-0.53553
C	1.62727	3.19078	-0.38698
C	2.43921	4.38075	-0.40344
C	3.75725	3.96301	-0.24476
C	3.7172	2.50701	-0.1291
N	2.41902	2.07613	-0.21773
C	4.97563	4.74961	-0.16238
C	5.18937	5.98737	-0.64529
C	1.92498	5.78334	-0.51518
Mg	1.78817	0.14726	-0.09529
N	3.73371	-0.53431	0.09425
C	4.85154	0.26613	0.11626
C	6.04891	-0.55858	0.2518
C	5.6257	-1.86252	0.30663
C	4.16692	-1.8339	0.20536
C	4.83537	1.66147	0.01544
C	-2.41124	2.45622	-2.25072
C	-3.3891	0.81157	1.04358

C	-4.76571	1.47661	0.94624
C	-5.46576	1.5326	2.29764
O	-4.96274	1.14149	3.32293
C	7.44757	-0.02484	0.31486
C	3.33026	-2.96735	0.20799
C	6.45322	-3.10524	0.47723
C	6.50435	-3.61169	1.93236
C	1.42235	-5.55191	0.20727
C	-3.35276	-2.44851	-1.31398
O	-3.12762	-1.91505	-2.38169
O	-2.22619	-4.9674	-0.06984
O	-4.48572	-3.12411	-1.04035
C	-5.40062	-3.26799	-2.13753
O	-6.71302	2.08146	2.36191
C	-7.46046	2.4596	1.18884
C	-8.16797	1.28766	0.56966
H	-0.26726	4.09883	-0.71363
H	5.80396	2.14896	0.03682
H	-3.32085	0.34316	-1.07225
H	3.81522	-3.93476	0.2892
H	-2.54119	2.96515	-0.15885
H	5.80785	4.26815	0.35102
H	-3.03313	-2.43531	0.78788
H	-2.0242	1.65653	-2.89172
H	-3.49163	2.53293	-2.42149
H	-1.94761	3.39791	-2.5626
H	-5.7391	-2.28891	-2.48858

H	-6.23721	-3.84646	-1.74498
H	-4.92389	-3.79751	-2.96639
H	0.89232	5.86429	-0.16025
H	1.94261	6.15494	-1.54988
H	2.53589	6.46987	0.08145
H	-3.51793	-0.20359	1.4316
H	-2.77454	1.33817	1.78242
H	-5.40304	0.93834	0.23322
H	-4.67772	2.50103	0.5576
H	1.96684	-5.765	1.13737
H	0.53343	-6.18717	0.1796
H	2.08039	-5.84797	-0.6211
H	-6.82997	2.97817	0.45859
H	-8.18784	3.18249	1.5738
H	7.60782	0.58411	1.21467
H	8.18605	-0.83154	0.32994
H	7.67359	0.61487	-0.5479
H	7.47534	-2.91621	0.12836
H	6.06073	-3.90229	-0.16802
H	7.10244	-4.52733	2.00527
H	6.95014	-2.85889	2.59153
H	5.49975	-3.82908	2.31088
H	6.14751	6.47863	-0.50329
H	4.44365	6.53568	-1.21078
C	-8.18592	1.02592	-0.73763
H	-8.71178	0.65373	1.26893
H	-7.65238	1.64677	-1.45513

H	-8.74684	0.18942	-1.1447

Table S17: Cartesian coordinates of ground state chlorophyll f (B3LYP functional with 6-31G (d) basis set)

C	-1.65734	-3.97826	0.0111
C	-0.23757	-3.64266	0.06188
C	-0.09715	-2.228	-0.01491
C	-1.31564	-1.52567	-0.10845
C	-2.42463	-2.57547	-0.06353
C	1.03762	-4.17964	0.1532
C	1.92713	-3.02718	0.12722
N	1.18481	-1.85289	0.02331
C	-1.41179	-0.1487	-0.22831
N	-0.30501	0.65907	-0.28446
C	-0.67458	1.95066	-0.51282
C	-2.17758	2.05946	-0.7477
C	-2.69589	0.66438	-0.30803
C	0.16305	3.0527	-0.5722
C	1.56859	3.09634	-0.43081
C	2.38446	4.29507	-0.46505
C	3.70795	3.89472	-0.26724
C	3.68071	2.45123	-0.15675
N	2.37011	2.0141	-0.24134
C	4.88036	4.76665	-0.19752
C	5.91623	4.62757	0.64332
C	1.93156	5.6722	-0.62125
Mg	1.7536	0.07794	-0.11101
N	3.71317	-0.58865	0.05052
C	4.82521	0.21186	0.02903
C	6.02485	-0.59839	0.12155
C	5.60812	-1.9087	0.20096
C	4.15559	-1.88484	0.15179
C	4.79577	1.61259	-0.06562
C	-2.49624	2.39026	-2.21691
C	-3.44376	0.68241	1.04454
C	-4.81328	1.3627	0.95378
C	-5.53915	1.35395	2.29266
O	-5.07042	0.87891	3.29878
C	7.4267	-0.0647	0.13384
C	3.32014	-3.01877	0.18643
C	6.45508	-3.14238	0.3512

C	6.60255	-3.60923	1.81257
C	1.42979	-5.62024	0.25397
C	-3.36249	-2.5746	-1.25937
O	-3.12912	-2.07787	-2.34197
O	-2.21117	-5.0585	0.0228
O	-4.50155	-3.22887	-0.96368
C	-5.42289	-3.39165	-2.05391
O	-6.77026	1.93579	2.36989
C	-7.48066	2.41759	1.21194
C	-8.21647	1.31515	0.5044
H	-0.30339	4.01499	-0.74949
H	5.75921	2.10926	-0.08908
H	-3.35289	0.2351	-1.07389
H	3.8132	-3.98319	0.26369
H	-2.59021	2.8543	-0.11434
H	4.88213	5.62028	-0.87404
H	-3.03984	-2.52283	0.843
H	-2.11046	1.61084	-2.88362
H	-3.57987	2.45855	-2.36989
H	-2.04825	3.34461	-2.51203
H	-5.74834	-2.41815	-2.4312
H	-6.26587	-3.9466	-1.64187
H	-4.95605	-3.95135	-2.86839
H	2.73879	6.43268	-0.56235
H	-3.58587	-0.33888	1.41126
H	-2.83117	1.18783	1.80001
H	-5.44217	0.86618	0.20339
H	-4.70953	2.40352	0.61771
H	1.99029	-5.82119	1.17592
H	0.54065	-6.25547	0.24936
H	2.0705	-5.92197	-0.58445
H	-6.81837	2.95932	0.52842
H	-8.18949	3.13967	1.63137
H	7.63158	0.51925	1.04098
H	8.16318	-0.87251	0.09456
H	7.61638	0.59494	-0.72196
H	7.45171	-2.95499	-0.06598
H	6.03252	-3.95952	-0.24762
H	7.21979	-4.51301	1.87294
H	7.0737	-2.83268	2.42493
H	5.62627	-3.83264	2.25656
H	6.74417	5.3303	0.62859
H	5.95691	3.83799	1.38761

C	-8.21429	1.13856	-0.81723
H	-8.79882	0.65895	1.15008
H	-7.64235	1.78375	-1.4817
H	-8.79634	0.35207	-1.28926
O	0.77667	6.03453	-0.80457

Table S18. Cartesian coordinates of excited state chlorophyll f (B3LYP functional with 6-31G (d) basis set)

C	-1.64616	-3.99406	0.00038
C	-0.23209	-3.65349	0.05482
C	-0.09292	-2.23767	-0.00989
C	-1.31957	-1.53577	-0.10075
C	-2.42028	-2.5914	-0.05948
C	1.04938	-4.18661	0.14467
C	1.93421	-3.03019	0.12866
N	1.18934	-1.85573	0.03384
C	-1.41476	-0.16277	-0.21665
N	-0.29392	0.65157	-0.26899
C	-0.67213	1.93417	-0.50874
C	-2.16954	2.04318	-0.74806
C	-2.69156	0.65355	-0.30167
C	0.16127	3.05517	-0.57112
C	1.55928	3.11355	-0.4275
C	2.36253	4.31564	-0.45889
C	3.6944	3.92094	-0.24886
C	3.67159	2.4691	-0.14057
N	2.37393	2.02373	-0.23195
C	4.84533	4.80506	-0.17961
C	5.97333	4.62686	0.5337

C	1.88074	5.67765	-0.61651
Mg	1.75787	0.07872	-0.09553
N	3.71172	-0.58185	0.06226
C	4.82236	0.22673	0.04016
C	6.0312	-0.5891	0.12795
C	5.61987	-1.89601	0.20415
C	4.16026	-1.87775	0.15834
C	4.79741	1.62132	-0.04733
C	-2.48494	2.36771	-2.22194
C	-3.44405	0.68334	1.05033
C	-4.81084	1.36798	0.95073
C	-5.53995	1.3672	2.28822
O	-5.07261	0.89726	3.29738
C	7.42678	-0.04481	0.13759
C	3.33346	-3.01892	0.18817
C	6.46421	-3.13124	0.34778
C	6.57994	-3.62669	1.80283
C	1.45187	-5.62343	0.23648
C	-3.36781	-2.58251	-1.24646
O	-3.15366	-2.0597	-2.32137
O	-2.19896	-5.07738	0.00255
O	-4.49248	-3.26193	-0.95417
C	-5.41925	-3.42275	-2.03995
O	-6.77019	1.95022	2.35954
C	-7.47836	2.4257	1.19729
C	-8.21068	1.31899	0.49288
H	-0.31516	4.01192	-0.75124

H	5.76079	2.11466	-0.08
H	-3.34874	0.2194	-1.06453
H	3.83049	-3.98116	0.25718
H	-2.5787	2.84677	-0.12281
H	4.75578	5.73129	-0.74612
H	-3.0274	-2.54898	0.85321
H	-2.10563	1.58009	-2.88236
H	-3.56814	2.44269	-2.37256
H	-2.03078	3.317	-2.52286
H	-5.76491	-2.44911	-2.39857
H	-6.24901	-3.99935	-1.63084
H	-4.94905	-3.96068	-2.86705
H	2.66511	6.45988	-0.54628
H	-3.59026	-0.3357	1.42095
H	-2.83124	1.19065	1.80421
H	-5.43882	0.86985	0.20077
H	-4.70286	2.40717	0.61076
H	2.01024	-5.8289	1.1597
H	0.56778	-6.26567	0.22252
H	2.10047	-5.91561	-0.60031
H	-6.81485	2.96538	0.51327
H	-8.18906	3.14869	1.6118
H	7.61886	0.55843	1.03506
H	8.17121	-0.84576	0.1165
H	7.61234	0.6035	-0.72819
H	7.46905	-2.93545	-0.04454
H	6.05295	-3.93569	-0.27596

H	7.19077	-4.53504	1.85719
H	7.0437	-2.8646	2.43846
H	5.5942	-3.85282	2.22366
H	6.7647	5.37001	0.51019
H	6.1262	3.77461	1.188
C	-8.20478	1.13657	-0.82796
H	-8.79392	0.66497	1.13994
H	-7.63229	1.7798	-1.49387
H	-8.7846	0.34728	-1.29802
O	0.71545	6.01283	-0.81342

In Tables S19 and S20 below, the atoms are numbered as in the Gaussian output files. To relate those numbers to the IUPAC numbering scheme of the chlorophyll structures: C1 and O42 are the carbon and oxygen of the 13^1-keto group; C40 and O41 are the carbonyl carbon and oxygen of the 13^3-ester group and O43 is the alkoxy oxygen of the 133-ester group; C33 and O34 are the carbonyl carbon and oxygen of the 17^3-ester group and O45 is the alkoxy oxygen of the 17^3-ester group; C22 and O85 are the carbon and oxygen of the 2^1-formyl group in chlorophyll f.

Table S19. Displacements of the atomic coordinates associated with carbonyl vibrational modes calculated for chlorophyll a in the ground and excited states using CAM-B3LYP method.

	Ground state chlorophyll a			Excited state chlorophyll a		
Frequency (cm^{-1})	1857.9697			1857.41		
Atom	X	Y	Z	X	Y	Z
C33	0.29	-0.23	0.72	0.30	-0.22	0.72
O34	-0.21	0.16	-0.45	-0.21	0.15	-0.45
O45	0.01	0.00	-0.03	0.01	-0.00	-0.03
Frequency (cm^{-1})	1833.1840			1828.18		
Atom	X	Y	Z	X	Y	Z

C1	0.22	0.38	0.01	0.13	0.24	0.01
C40	-0.12	-0.17	0.62	-0.14	-0.21	0.68
O41	0.10	0.12	-0.38	0.11	0.14	-0.43
O42	-0.13	-0.24	-0.01	-0.08	-0.15	-0.00
O43	0.02	0.01	-0.04	0.02	0.02	-0.04
Frequency (cm^{-1})		1816.0523			1806.18	
Atom	X	Y	Z	X	Y	Z
C1	0.37	0.55	0.02	0.42	0.64	0.02
C40	0.13	0.14	-0.41	0.10	0.11	-0.25
O41	-0.07	-0.08	0.26	-0.05	-0.06	0.16
O42	-0.20	-0.37	-0.01	-0.24	-0.42	-0.01
O43	-0.03	-0.02	0.02	-0.02	-0.02	0.02

Table S20. Displacements of the atomic coordinates associated with carbonyl vibrational modes calculated for chlorophyll f in the ground and excited states using CAM-B3LYP method.

	Ground state chlorophyll f			Excited state chlorophyll f		
Frequency (cm^{-1})		1856.57			1856.55	
Atom	X	Y	Z	X	Y	Z
C33	0.28	-0.27	0.71	0.28	-0.26	0.71
O34	-0.20	0.18	-0.45	-0.20	0.18	-0.45
O45	0.01	0.00	-0.03	0.01	0.00	-0.03
Frequency (cm^{-1})		1837.32			1829.35	
Atom	X	Y	Z	X	Y	Z
C1	0.28	0.49	0.00	0.15	0.28	0.00

C40	-0.10	-0.12	0.54	-0.13	-0.19	0.67
O41	0.08	0.09	-0.33	0.10	0.13	-0.42
O42	-0.17	-0.32	0.00	-0.09	-0.18	-0.00
O43	0.01	0.01	0.03	0.02	0.02	-0.04
Frequency (cm^{-1})		1821.06			1810.09	
Atom	X	Y	Z	X	Y	Z
C1	-0.31	-0.46	-0.00	0.40	0.62	0.01
C40	-0.16	-0.15	0.52	0.11	0.12	-0.30
O41	0.09	0.09	-0.33	-0.05	-0.06	0.19
O42	0.17	0.31	-0.00	-0.22	-0.41	-0.00
O43	0.03	0.02	-0.03	-0.03	-0.02	0.02
Frequency (cm^{-1})		1785.68			1769.05	
Atom	X	Y	Z	X	Y	Z
C22	0.64	-0.27	0.11	0.62	-0.25	0.11
O85	-0.42	0.14	-0.07	-0.41	0.13	-0.07
